# Neuropeptides and Microglial Activation in Inflammation, Pain, and Neurodegenerative Diseases

**DOI:** 10.1155/2017/5048616

**Published:** 2017-01-05

**Authors:** Lila Carniglia, Delia Ramírez, Daniela Durand, Julieta Saba, Juan Turati, Carla Caruso, Teresa N. Scimonelli, Mercedes Lasaga

**Affiliations:** ^1^INBIOMED (Instituto de Investigaciones Biomédicas), UBA-CONICET, Facultad de Medicina, Universidad de Buenos Aires, Paraguay 2155, Ciudad Autónoma de Buenos Aires, Argentina; ^2^IFEC-CONICET, Depto. Farmacología, Facultad de Ciencias Químicas, Universidad Nacional de Córdoba, Córdoba, Argentina

## Abstract

Microglial cells are responsible for immune surveillance within the CNS. They respond to noxious stimuli by releasing inflammatory mediators and mounting an effective inflammatory response. This is followed by release of anti-inflammatory mediators and resolution of the inflammatory response. Alterations to this delicate process may lead to tissue damage, neuroinflammation, and neurodegeneration. Chronic pain, such as inflammatory or neuropathic pain, is accompanied by neuroimmune activation, and the role of glial cells in the initiation and maintenance of chronic pain has been the subject of increasing research over the last two decades. Neuropeptides are small amino acidic molecules with the ability to regulate neuronal activity and thereby affect various functions such as thermoregulation, reproductive behavior, food and water intake, and circadian rhythms. Neuropeptides can also affect inflammatory responses and pain sensitivity by modulating the activity of glial cells. The last decade has witnessed growing interest in the study of microglial activation and its modulation by neuropeptides in the hope of developing new therapeutics for treating neurodegenerative diseases and chronic pain. This review summarizes the current literature on the way in which several neuropeptides modulate microglial activity and response to tissue damage and how this modulation may affect pain sensitivity.

## 1. Introduction

Microglial cells are the resident macrophage-like cells of the central nervous system (CNS). They are in charge of immune surveillance and of sampling the microenvironment through their numerous cellular processes. When faced with an insult or pathogenic agent they rapidly respond by developing a classic proinflammatory program (termed M1), releasing inflammatory mediators such as tumour necrosis factor- (TNF-) *α*, Interleukin- (IL-) 1*β*, IL-12, reactive oxygen species (ROS), nitric oxide (NO), prostaglandins (PGs), and chemokines that help recruit other immune cells to the injured zone and amplify the inflammatory response. Once the noxious stimulus has been dealt with, it is crucial that the inflammatory response be dampened and resolved; this is achieved by active redirection of microglial phenotype towards an alternative immunomodulatory or acquired deactivation profile (termed M2), characterized by release of anti-inflammatory cytokines such as transforming growth factor- (TGF-) *β* and IL-10, and expression of Arginase-1 (AG1) [1]. These immunomodulatory mediators inhibit the release of proinflammatory factors from immune and nonimmune cells and promote tissue regeneration, thereby facilitating the return to homeostasis. When the resolution phase of the inflammatory response is altered, excessive damage to the affected tissue may ensue, potentially leading to cell death and neurodegeneration. In fact, microglial proinflammatory activation has been implicated in the pathology of many neurodegenerative disorders such as Parkinson's disease (PD) [2], Alzheimer's disease (AD) [3], multiple sclerosis (MS), and AIDS dementia [4] where neuronal damage occurs as a consequence of a prolonged proinflammatory response and more recently, in several neuropsychiatric conditions [5]. Thus, the modulation of microglial activation is of great importance in the context of inflammatory and degenerative diseases of the CNS.

Over the last two decades, increasing attention has been brought to the role of glial cells in the development and maintenance of chronic pain, cumulative evidence suggesting that chronic pain could be the result of dysregulated glial activity [6]. Mediators such as proinflammatory cytokines, chemokines, PGE_2_, and NO produced mainly by microglial cells and by other nonneuronal cells of the nervous and immune systems are known to contribute to pain hypersensitivity by activating nociceptive neurons in the CNS and in the peripheral nervous system (PNS) [7]. Concordantly, changes in morphological features and expression of molecular markers characteristic of an activated microglial phenotype have been observed in different animal models of nerve injury and pain [8], strongly suggesting reactive microglia might be involved in these pathological processes of the nervous system. In particular, many studies have demonstrated a central role for microglial p38 mitogen-activated protein kinase (MAPK) activation in the pathogenesis of neuropathic pain [7]. The role of this kinase in microglial signaling is pivotal since it can be activated by multiple microglial receptors, and it also regulates the synthesis of many inflammatory mediators associated with pain facilitation. Moreover, p38 MAPK is selectively activated in spinal microglial cells after spinal nerve ligation (SNL), and administration of a p38 MAPK inhibitor significantly attenuates allodynia [7], underscoring its role in nociception. Another well-recognized mediator of microglial-neuron communication in neuropathic pain transmission is brain-derived neurotrophic factor (BDNF), which is induced and released from microglial cells upon ATP stimulation, and mediates the depolarizing shift in the anion reversal potential in spinal neurons underlying neuropathic pain [9]. To further underscore the role of microglia in nociception, administration of the microglial inhibitor minocycline has been shown to attenuate pain hypersensitivity in models of pain facilitation [10], spinal cord injury (SCI) [11], burn injury-induced pain [12], SNL [13], inflammation-evoked hyperalgesia [14], and chronic constriction injury (CCI) [15], among others. Altogether, evidence suggests that modulators of microglial activation could potentially be employed as antinociceptive agents in pain management.

Neuropeptides are small amino acidic molecules produced mainly, though not exclusively, by cells of the nervous system. Typically, they have the capacity to regulate neuronal activity and may affect a great variety of central and peripheral functions, such as thermoregulation, reproductive behavior, food and water intake, and circadian rhythms [16]. Neuropeptides are also major regulators of immune and inflammatory responses; they affect pain sensitivity and are known to play an important role in neurogenesis, acting not only on neuronal cells but also on glial cells. Microglial cells express a variety of neuropeptide and neurotransmitter receptors which modulate microglial function [17], and the last decade has witnessed growing interest in the study of microglial activation, their role in neuroinflammation and neurodegeneration, and their modulation by neuropeptides.

**Figure 1:**
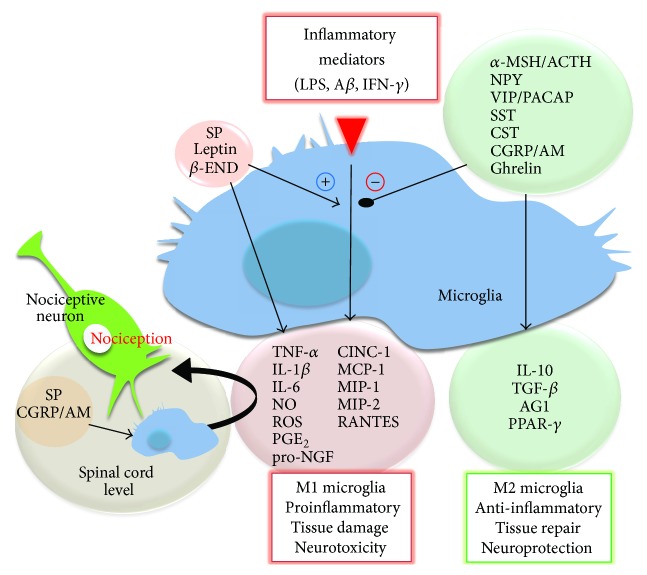
Neuropeptide modulation of microglial activation. In the presence of noxious stimuli such as LPS, A*β*, and IFN-*γ* or in the context of neuroinflammation or tissue damage, microglial cells produce a plethora of inflammatory mediators that promote and perpetuate the inflammatory response, potentially leading to neurodegeneration. Neuropeptides, acting through specific receptors present in microglial cells, are able to modulate microglial response and inhibit the release of inflammatory mediators while favoring development of an alternative activation program. On the other hand, neuropeptides such as SP, *β*-END, and leptin exert proinflammatory actions on microglial cells and may even potentiate the response to noxious stimuli. In experimental models of pain, certain neuropeptides such as SP, CGRP, and AM have been suggested to increase pain hypersensitivity partly by promoting proinflammatory activation of spinal cord microglia.

Here, we review the role of neuropeptides in the modulation of microglial function (Figure 1). We have selected neuropeptides for which microglial cells are known to express specific receptors (Table 1), which might therefore directly affect microglial activity and influence processes such as inflammation, neurodegeneration, and pain sensitivity. Although the main focus is set on microglial cells, some data obtained in macrophages is provided in order to discuss similarities and differences between these cell types. It is important to bear in mind that microglial cells comprise a very heterogeneous population, and this heterogeneity also applies to their ability to respond to neuropeptides; in vitro studies using isolated microglia have demonstrated that only a fraction of the whole population actually responds to a certain neuropeptide and that this fraction can be altered depending on the activation state of the cells [18]. Consequently, caution should be taken when interpreting data from isolated microglia, and more importantly, when comparing the behavior of primary microglia to that of peripheral macrophages or to that of a microglia/macrophage cell line.

## 2. POMC-Derived Peptides

Within the CNS, the proopiomelanocortin (POMC) gene is mainly expressed in neurons from the arcuate nucleus (ARC) of the hypothalamus which project into the paraventricular nucleus, lateral hypothalamus, and other regions of the brain such as amygdala, cortex, hippocampus, medulla, mesencephalon, and spinal cord [19]. Through the action of tissue-specific prohormone convertases, it is posttranslationally processed to yield adrenocorticotropic hormone (ACTH), *α*-melanocyte-stimulating hormone (*α*-MSH), *β*-MSH, and *γ*-MSH, collectively known as melanocortins, and the endogenous opioid *β*-endorphin (*β*-END). The melanocortin system is involved in the regulation of a great variety of functions such as food intake and energy expenditure, sexual behavior, exocrine gland secretion, fever control, pigmentation, learning, and memory [20, 21]. This system is also tightly linked to control of inflammation, both centrally and peripherally, by exerting anti-inflammatory actions on cells of the immune system including lymphocytes, monocytes, and macrophages, as well as nonimmune cells such as melanocytes, keratinocytes, and adipocytes, among others [22]. Melanocortins may also regulate macrophage differentiation into tissue-specific cells such as osteoclasts, Kupffer cells, and microglia [23], further highlighting the role of these neuropeptides as modulators of immunity.

Despite the long recognized anti-inflammatory properties of melanocortins, little is known about their direct influence on microglial cells, which are the main component of innate immunity in the CNS. In addition, most of the studies on the influence of POMC products on microglia used cell lines instead of primary microglia and focused on the effect of ACTH and *α*-MSH, along with several synthetic analogs of these peptides, whereas the effects of *γ*-MSH, *β*-MSH, and *β*-END have been studied less. Almost two decades ago, *α*-MSH was demonstrated to inhibit brain production of TNF-*α* in a mouse model of brain inflammation caused by bacterial lipopolysaccharide (LPS) injection [24]. The authors suggested that *α*-MSH was acting through the activation of MC1R present on astrocytes and microglia, the main producers of TNF-*α* within the brain. A year later, ACTH, *α*-MSH, and *α*-MSH COOH-terminal tripeptide (*α*-MSH_11–13_) were shown to inhibit LPS + Interferon- (IFN-) *γ*-induced TNF-*α*, IL-6, and NO release in N9 microglial cells, most likely by a mechanism involving cAMP production [25]. The authors also demonstrated that microglial cells release *α*-MSH upon stimulation with LPS + IFN-*γ*, thereby creating an autocrine anti-inflammatory loop. This finding placed microglial cells in the group of nonneuronal immune cells expressing POMC mRNA and producing *α*-MSH as an autoregulatory factor, similar to what had been previously demonstrated in RAW 264.7 murine macrophages [26] and in human THP-1 myelomonocytic cells [27]. Subsequently, *α*-MSH and *α*-MSH_11–13_ were also demonstrated to reduce the release of TNF-*α* and NO and the expression of TNF-*α* and inducible nitric oxide synthase (iNOS) induced by costimulation of N9 cells with Amyloid-*β* (A*β*) and IFN-*γ* [28], further supporting its role as an anti-inflammatory agent. Since then, melanocortins have proven to be anti-inflammatory and neuroprotective in vivo in several experimental models of nervous tissue damage, including brain ischemia [29–31] and traumatic brain injury, where neuroprotection exerted by *α*-MSH_(11–13)_ was associated with decreased microglial activation [32], suggesting a contribution of local immunomodulation to the overall neuroprotective effect.

Melanocortins exert their actions through MCRs of which five subtypes have been described up to now (MCR1 to MCR5). These are G protein-coupled receptors (GPCRs) with seven transmembrane domains, all positively coupled with adenylate cyclase (AC) [33]. Nonetheless, cAMP-independent pathways have also been shown to mediate MCR signaling (reviewed in [34]). In the brain, the most abundant MCR subtypes are MC3R and MC4R [35, 36]. Expression of MC1R has been detected in RAW 264.7 cells [26], human monocytes [37], and murine macrophages [38]. MC3R expression was found in murine and rat macrophages [39, 40] and in RAW 264.7 cells [41] and expression of MC1R, MC3R, and MC5R was detected in THP-1 cells [27, 42]. Human monocytes express mRNA for MC1R, MC2R, MC3R, and MC5R [43]. MC4R, however, does not appear to be expressed in mouse macrophages [39] and RAW 264.7 cells [44]. On the other hand, MCR expression in microglial cells has not been thoroughly examined. Lindberg et al. showed that the human microglial cell line CHME-3 expresses MC1, MC3, MC4, and MC5 receptors and that *α*-MSH increases basal IL-6 secretion in these cells [45]. The authors suggested that this effect is mediated by either MC3R or MC5R, since agouti, an endogenous antagonist of the melanocortin system which has greater affinity for MC1R and MC4R than for other MCRs [46], did not block *α*-MSH induction of IL-6 but instead enhanced it. Contrary to what was described in macrophages, our group found MC4R expression in rat primary microglia, whereas expression of MC1R, MC3R, and MC5R was not detected by RT-PCR in these cells [47]. These data suggest that a certain degree of heterogeneity exists regarding MCR expression between microglia and peripheral macrophages and when comparing primary and transformed cell systems.

In RAW 264.7 macrophages, ACTH and melanotan II (MTII, a MC3/4R agonist) induce rapid cAMP accumulation and p38 MAPK phosphorylation but do not alter extracellular signal-regulated kinases (ERK)1/2 and c-Jun N-terminal kinase (JNK) phosphorylation [41]. ACTH and MTII also stimulate IL-10 production through a protein kinase A- (PKA-) dependent mechanism [41]. The rapid increase in cAMP has also been observed in N9 cells and was proposed to mediate anti-inflammatory actions of melanocortins [25]. In CHME3 cells Lindberg and colleagues found that PKA was not involved in *α*-MSH-induced IL-6 release, but when *α*-MSH was combined with a PKA inhibitor, this led to a decrease in cell viability [45], indicating that certain mechanisms triggered by *α*-MSH do involve PKA activity in microglia. Our group found that [Nle_4_, D-Phe_7_]-*α*-MSH (NDP-MSH), a synthetic analog of *α*-MSH, induced a marked decrease in peroxisome proliferator-activated receptor- (PPAR-) *β* protein expression, which was prevented by inhibition of ERK1/2, thereby indicating a role for this kinase in melanocortin signaling in primary microglial cells [47].

*α*-MSH was shown to suppress Toll-like receptor (TLR) 4-induced (but not TLR2-induced) NO production in the macrophage cell line J774, where it also promoted IL-1 receptor associated kinase- (IRAK-) M binding to IRAK-1, thereby suppressing TLR4-dependent response to LPS at a very proximal stage in the signaling cascade [48]. NDP-MSH reduces TLR4 gene expression in rat microglial cells [49], and *α*-MSH reduces expression of the LPS coreceptor CD14 in THP-1-derived macrophages [50]. Evidence also indicates that melanocortins inhibit NF-*κ*B activity by preventing I*κ*B degradation and p65 nuclear translocation [51]. In the U-937 monocytic cell line these effects appear to be mediated by the cAMP-PKA pathway [51], whereas in RAW 264.7 cells inhibition of NF-*κ*B by *α*-MSH seems to be a cAMP-independent mechanism [52]. Concordantly, NDP-MSH prevented LPS-induced p65 and c-Rel translocation to the nucleus in primary cultured rat microglial cells [49].

The importance of *α*-MSH in maintaining immunosuppression in the ocular microenvironment was identified long ago [53]. More recently, a study showed that *α*-MSH produced in the healthy retina is necessary for induction of AG1 expression in retinal microglial cells [54]. Since AG1 is a marker of alternatively activated M2 microglia and macrophages [1], *α*-MSH, in cooperation with neuropeptide Y (NPY) and other local neuropeptides, might be responsible for maintaining ocular immune privilege by alternatively activating local macrophages and microglia towards an immunosuppressive or tolerogenic phenotype [54]. On a similar note, our group studied the effects of NDP-MSH on rat microglial cells and found that it induces release of the anti-inflammatory cytokine IL-10 and expression of PPAR-*γ* [47] and of AG1 [49], all markers of alternative activation [1], further reinforcing a role for this neuropeptide in promoting an alternative activation program in microglial cells.

Phagocytosis of debris, pathogens, or apoptotic cells is an important part of the resolutory phase of inflammation. It is generally regarded as a beneficial phenomenon and its alteration has been linked to autoimmune disorders and neurodegenerative diseases [75]. However, phagocytosis can also activate the respiratory burst and increase generation of ROS, which may cause neurotoxicity [75]. In fact, both classical M1 (driven by IFN-*γ*) and alternative M2 (driven by IL-4 and IL-13) microglial activation paradigms have been found to induce phagocytosis of apoptotic cells and of purified myelin, respectively [76, 77]. In murine primary macrophages, the MCR agonist AP124 promotes phagocytosis of zymosan particles and of apoptotic neutrophils acting mainly through MC3R [78]. However, treatment of RAW 264.7 cells with *α*-MSH reduces phagocytosis of apoptotic cells [79] and* E. coli* bioparticles, and when combined with NPY it also reduces phagocytosis of gram-positive bioparticles [80]. Our group found that treatment of primary rat microglia with NDP-MSH does not affect basal or LPS-induced phagocytosis of latex beads; however, NDP-MSH inhibits phagocytosis induced by the TLR2 agonist Pam_3_CSK_4_ in microglial cells [49]. It is clear that the effects of neuropeptides on phagocytosis are highly dependent on variables such as the cell type involved, the stimulus, and the system of choice for studying phagocytosis (i.e., latex beads, opsonized versus unopsonized bioparticles, and apoptotic cells).

Evidence indicates that POMC-derived peptides play an important role in nociception and that at the spinal cord level melanocortin and opioid systems interact and cooperate with each other in pain perception, in that MCR activation seems to generally increase pain sensitivity whereas activation of opioid receptors by *β*-END induces analgesia [81]. Expression of POMC, *β*-END, ACTH, *α*-MSH, the endogenous antagonist Agouti-Related Protein (AgRP), and MC4R has been detected in areas of the spinal cord involved in nociception, underscoring the role of this system in nociceptive transmission [82–85]. Concordantly, several studies have shown that antagonism of the melanocortin system reduces neuropathic pain [86–88] and prevents morphine withdrawal hyperalgesia [89]. It has been established that spinal cord microglial cells play a crucial role in neuropathic pain by releasing mediators that activate nociceptive neurons [90]. However, they can also be a source of *β*-END and release it in response to diverse stimuli, such as corticotrophin-releasing hormone (CRH) or noradrenaline stimulation [91], and upon glucagon-like peptide-1 (GLP-1) receptor activation [92]. Induction of *β*-END by a GLP-1 receptor agonist was found to be mediated by a p38 MAPK-dependent mechanism in spinal dorsal horn microglia [93]. Microglial *β*-END, in turn, mediates GLP-1 antinociceptive effects in pain hypersensitivity states by activating opioid receptors located on neurons [92]. In addition, *β*-END (and other opioid receptor ligands) may act directly on microglia, although little is known about direct effects of *β*-END on these cells. Of the opioid receptors so far identified, microglia express *μ*- and *κ*-receptors, but not *δ*-receptors, and an additional opioid receptor-independent pathway may also exist for endogenous opioid receptor ligands [17, 55–57]. However, activation of the GLP-1 receptor/*β*-END pathway does not inhibit proinflammatory cytokine production in cultured microglia stimulated by LPS [94]. On the contrary, *β*-END has been shown to directly enhance basal and GP-120-induced TNF-*α*, IL-1*β*, and IL-6 release in brain perivascular microglia, where it also enhances replication of the human immunodeficiency virus (HIV), effects that are blocked by an opioid receptor antagonist [95]. Concordantly, prolonged treatment with morphine induces microglial activation and upregulation of proinflammatory cytokines such as IL-6, TNF-*α*, and IL-1*β* in the rat spinal dorsal horn [96, 97]. Since these inflammatory mediators may in turn activate spinal nociceptive neurons and enhance pain sensitivity [7], the direct effect of opioids on microglial cells may partially account for the development of tolerance to long-term opioid treatment. This notion is supported by evidence showing that development of tolerance is mediated by activation of microglial p38 MAPK [98] and that treatment with the microglial inhibitor minocycline prevents morphine-induced tolerance [99].

## 3. NPY

NPY is a 36-amino acid neuropeptide derived from its precursor pre-pro-NPY. It belongs to the family of pancreatic peptides together with peptide YY (PYY) and pancreatic polypeptide (PP) and is widely distributed within the CNS and PNS. The physiological functions of NPY are varied and include regulation of blood pressure, circadian rhythms, feeding behavior, anxiety, and memory [100].

In mammals, five subtypes of Y receptors have been described (Y_1_R, Y_2_R, Y_4_R, Y_5_R, and Y_6_R). Y_1_R, Y_2_R, and Y_5_R are the preferential receptors for NPY and PYY, Y_4_R binds PP, and Y_6_R is a pseudogene in humans that has been lost in rats (reviewed in [101]). They are all GPCRs whose main signaling pathway involves coupling with G_*i*/0_ proteins and inhibition of cAMP production, although other possible signaling pathways include intracellular calcium mobilization, modulation of Ca^2+^ and K^+^ channel conductance, activation of PKC, PKA, phospholipase (PL) A2, MAPKs, guanylyl cyclase, and phosphatidylinositol 3-kinase (PI3K) [102].

Expression of NPY and of Y_1_R, Y_2_R, and Y_5_R has been detected in N9 murine microglia [58]. In these cells, gene and protein expression of NPY and Y_1_R increase with LPS treatment [58], suggesting the neuropeptide might have a regulatory function on LPS-induced microglial activation. In accordance with a proposed anti-inflammatory role, NPY inhibits LPS-induced iNOS expression, NO, and IL-1*β* release, and it also inhibits IL-1*β*-induced cell motility, iNOS expression, NO production, and NF-*κ*B activation in N9 cells [58, 103]. NPY protected N9 microglia from methamphetamine- (METH-) induced apoptosis through a mechanism partially dependent on Y_2_R and independent from Y_1_R, and an Y_2_R agonist reduced microglial CD11b immunoreactivity in hippocampal organotypic cultures incubated with METH, thereby suggesting a protective role for NPY via Y_2_R in METH-induced microglial dysfunction [104]. Inflammatory cytokines released by glial cells are proposed to mediate local increase in neuronal excitability and apoptotic death in experimental models of seizures [105]. Concordantly, conditioned medium from LPS-treated rat microglial cells (with elevated levels of IL-1*β* and TNF-*α*) increase neuronal NMDA current, which when excessive may cause excitotoxic neuronal injury [106]. In the same study, cotreatment of microglial cells with LPS + NPY prevented the LPS-induced increase in IL-1*β* and TNF-*α* production through a mechanism involving Y_1_R. Furthermore, the effect of microglial LPS-conditioned medium on NMDA current was not observed when neurons were incubated with medium from LPS + NPY cotreated microglia, suggesting that the anti-inflammatory effect of NPY on microglial cells might in turn be neuroprotective by preventing NMDA receptor hyperactivation [106].

NPY seems to play a special role in retinal physiology [102]. It is expressed in different neuronal and glial cells of the retina, including microglia [107]. The NPY receptors Y_1_R and Y_2_R are also expressed in retinal microglial cells [59], suggesting a possible modulatory role for this neuropeptide in inflammatory retinal dysfunctions. Moreover, it has been proposed that NPY released by the ocular posterior chamber and derived mainly from the retinal pigment epithelial cells promotes (in cooperation with *α*-MSH) the development of myeloid suppressor cell-like characteristics in retinal microglia and in migrating macrophages [54]. In addition, NPY modulates phagocytosis in professional phagocytes and antigen-presenting cells [108]. In RAW 264.7 cells, NPY suppresses phagocytosis of unopsonized bioparticles, phagolysosome activation, and Fc receptor-mediated generation of ROS [80]. Treatment of microglial cells with NPY inhibits LPS-induced actin cytoskeleton reorganization and phagocytosis of opsonized latex beads acting through Y_1_R [109]. NPY also decreases LPS- and IL-1*β*-induced Fc receptor expression and prevents activation of p38 MAPK and HSP27 triggered by LPS in N9 cells [109]. The ability of NPY to act on microglial cells and to negatively regulate phagocytosis further supports a role for this neuropeptide (together with *α*-MSH) in maintaining the immune-privileged ocular microenvironment.

The NPY system is a key element in pain modulation. Evidence shows that intrathecal administration of NPY reduces behavioral signs of inflammatory and neuropathic pain, acting at spinal Y_1_R and Y_2_R [110, 111]. Considering that NPY decreases microglial proinflammatory activation, it would be interesting to study the potential role of microglia as mediators of NPY analgesic effects, an area which, to our knowledge, has not yet been addressed.

## 4. VIP and PACAP

The Vasoactive Intestinal Peptide (VIP) is a 28-amino acid polypeptide discovered in 1970 in intestinal extracts, able to induce vasodilation [112]. VIP is an immunoregulatory neuropeptide produced by neurons, endocrine, and immune cells, widely distributed in the CNS and PNS, and also found in heart, lung, thyroid, kidney, urinary and gastrointestinal tracts, genital organs, and the immune system [113]. VIP is structurally related to the secretin/glucagon family of peptides and hormones, sharing 70% sequence identity with the neuropeptide pituitary adenylate cyclase-activating polypeptide (PACAP) [114]. The precursor molecule pre-pro-VIP is processed into mature VIP and the related peptide C-terminal methionine amide (PHM) in humans and peptide with N-terminal histidine and C-terminal isoleucine amide (PHI) in other mammalian species [115].

PACAP was discovered nearly two decades later as a 38-amino acid hypothalamic neuropeptide that stimulated AC activity and increased cAMP levels in pituitary cells [116]. The sequence of PACAP-38 contains an internal cleavage site giving rise to a 27-amino acid fragment corresponding to the N-terminal portion of PACAP-38, termed PACAP-27, which is also capable of inducing AC activity in pituitary cells [117]. In the CNS, the predominant form is PACAP-38, expressed mainly in the hypothalamus but also widely distributed in several extrahypothalamic regions. In the periphery, PACAP-38 is also the predominant form and has been found in numerous organs and tissues including the immune system [117].

Following the discovery of PACAP, it was found that VIP and PACAP act as endogenous ligands for the same receptors, which are three heterotrimeric GPCRs: PAC1, VPAC1 (or VIPR1), and VPAC2 (or VIPR2), each with a distinct expression pattern. PAC1 has much greater affinity for PACAP than for VIP, whereas VPAC1 and VPAC2 can bind PACAP and VIP with comparable affinity [118]. Expression of PAC1 is highest in the CNS, particularly in the thalamus, hypothalamus, hippocampus, olfactory bulb, and cerebellum. VPAC1 is also widely distributed in the CNS, particularly in the cerebral cortex and hippocampus, and in several peripheral organs [118]. VPAC2 expression has been detected in the thalamus, suprachiasmatic nucleus, hippocampus, brainstem, and dorsal root ganglia, and in some peripheral tissues such as smooth muscle of the cardiovascular, gastrointestinal, and reproductive systems [118]. These receptors are coupled primarily with Gs and activate AC and PKA, although VPAC/PAC1 activation has also been reported to activate or inhibit several other signaling pathways [119, 120].

VIP and PACAP proved to be neuroprotective in various models of nervous tissue damage [121]. Since microglial cells are the main source of inflammatory mediators in the CNS, it has been suggested that VIP and PACAP might be exerting their protective effects in part by acting directly on microglial cells as microglia-deactivating factors. Expression of VPAC1 and PAC1 (but not VPAC2) was detected in rat microglia, where these receptors mediate VIP and PACAP inhibition of LPS-induced TNF-*α* production in a cAMP-dependent manner [60]. Similarly, Delgado and colleagues found expression of VPAC1 and PAC1, but not VPAC2, in murine microglia, where VIP and PACAP inhibit LPS-stimulated production of the CXC chemokines macrophage-inflammatory protein- (MIP-) 2 and KC, and of the CC chemokines MIP-1*α*, MIP-1*β*, macrophage chemoattractant peptide (MCP)-1, and RANTES, and inhibit chemotactic activity of peripheral leukocytes [61]. VIP and PACAP also prevent the production of LPS-induced TNF-*α*, IL-1*β*, IL-6, and NO in murine microglia [122]. These effects are attained through activation of VPAC1, induction of cAMP production, and inhibition of NF-*κ*B [61, 122]. The mechanism for VIP and PACAP inhibition of NF-*κ*B in activated microglial cells involves a reduction in p65 binding to the coactivator CREB-binding protein (CBP) and parallel stimulation of CREB phosphorylation, thereby promoting CREB/CBP complex formation instead of p65/CBP interaction [123] and ultimately leading to reduced transcription of classic proinflammatory NF-*κ*B-regulated genes. VIP inhibits cyclooxygenase- (COX-) 2 expression and PGE_2_ production in LPS- and LPS + IFN-*γ*-treated murine microglial cells [124]. Activation of VPAC1-cAMP signaling by VIP and PACAP was also found to inhibit the MEKK1/MEK4/JNK pathway and subsequent binding of the transcription factor AP-1 in LPS-stimulated microglial cells [125]. PACAP was also shown to prevent LPS-induced BV-2 microglial activation by inducing cAMP production and inhibiting p38 MAPK signaling pathway [126]. This neuropeptide also attenuated microglial hypoxia-induced TNF-*α*, iNOS, and NO production and p38 MAPK activation and prevented neuronal death caused by microglial neurotoxicity [127]. In IFN-*γ*-treated microglial cells, activation of VPAC1 by VIP and PACAP inhibits expression of IFN-*γ*-inducible protein- (IP-) 10, iNOS, and CD40 through inhibition of the IFN-*γ*-induced JAK1-2/STAT1 pathway [128]. Another study demonstrated that PACAP and VIP downregulate mRNA and surface protein expression of costimulatory molecules CD40 and B7-2 in activated microglial cells. In this study, the effect of PACAP on CD40 was mediated by VPAC1 activation and, at least in part, by PACAP-induced IL-10 [129]. Altogether, evidence indicates a strong anti-inflammatory and immunosuppressive role for VIP and PACAP in microglial cells, mainly through VPAC1 and PAC1 activation.

Concordant with these neuroprotective and anti-inflammatory actions, treatment with VIP or PACAP protected mice from lethal endotoxemia, the protective effect being attributed to inhibition of endotoxin-induced TNF-*α* and IL-6 production [130]. Apart from inhibiting proinflammatory mediators, VIP and PACAP were found to rapidly increase IL-10 production in LPS-treated macrophages, primarily through activation of VPAC1, elevation of cAMP and increased CRE binding activity [131], and the stimulatory effect of VIP and PACAP on IL-10 production was also observed in vivo in LPS-treated mice [131].

A recent study suggested that endogenous PACAP may be neuroprotective during seizure by acting on microglial cells and inducing their polarization towards an alternative M2 phenotype [132]. VIP and PACAP also exert beneficial effects in several in vitro and in vivo models of neuroinflammation and neurodegeneration. Treatment with VIP prevented several features of mechanically induced brain trauma, such as microglial activation, proinflammatory cytokine secretion, leukocyte infiltration, and neurodegeneration [133]. In a model of brain focal ischemia, delayed intracerebroventricular delivery of PACAP-producing stem cells promoted fast and stable functional recovery which correlated with reduced inflammatory response and increased number of AG1^+^ microglia, further supporting a role for PACAP in polarizing microglia towards an M2 neuroprotective phenotype [134]. In an in vitro model of ischemic damage by oxygen and glucose deprivation (OGD) and reoxygenation, pretreatment of BV-2 cells with PACAP alleviated hypoxic injury by preventing TLR4/MyD88/NF-*κ*B signaling and decreasing proinflammatory cytokine levels and BV-2 apoptosis [135]. In a mouse model of glaucomatous retinal damage by intravitreal NMDA injection, PACAP-38 prevented NMDA-induced cell death in the ganglion cell layer, including retinal ganglion cells and Iba1^+^ cells, presumably through a mechanism dependent on IL-10 release by retinal microglia/macrophages [136]. PACAP also increased mRNA levels of the anti-inflammatory mediators TGF-*β* and IL-10, which partially colocalized with Iba1^+^ cells and CD11b^+^ cells, providing additional evidence of PACAP inducing an acquired deactivation profile in microglia/macrophages.

Delgado and colleagues found that VIP inhibits A*β*-induced microglial activation, production of TNF-*α*, IL-1*β*, and NO, and activation of NADPH-oxidase and reduces neuronal death induced by fibrillar A*β*_1–42_ in murine neuron-microglia cocultures [137]. These effects were mediated by the VPAC1-cAMP-PKA signaling pathway and by inhibition of p38, ERK1/2, and NF-*κ*B signaling cascades [137]. VIP also inhibited TNF-*α* and NO production in mouse primary microglia stimulated with A*β*_42_ + low dose IFN-*γ*, promoted microglial phagocytosis of A*β*_1–42_ through a PKC-dependent mechanism, and reduced A*β* deposits in the hippocampus of transgenic PS1/APP mice, a model of AD [138], suggesting a protective role for VIP in AD through modulation of microglial function.

PACAP-38 was shown to inhibit NADPH oxidase activity and ROS production in microglial cells. This mechanism appears to be involved in PACAP-induced neuroprotection and is not mediated by classic VPAC or PAC1 receptors [139]. This effect is of particular importance in PD, since dopaminergic neurons in the substantia nigra are highly vulnerable to oxidative stress [140]. In mice treated with the neurotoxin 1-methyl-4-phenyl-1,2,3,6-tetrahydropyridine (MPTP), a model of PD, administration of VIP into the substantia nigra inhibited microglial activation and expression of the cytotoxic mediators TNF-*α*, IL-1*β*, iNOS, and NADPH-oxidase and reduced nigrostriatal nerve fiber loss and dopaminergic neuronal degeneration in the ipsilateral substantia nigra pars compacta [141]. PACAP and a VPAC1 agonist (but not a VPAC2 agonist) also prevented MPTP-induced microglial activation and dopaminergic neuronal degeneration with a potency similar to VIP, suggesting that the protective actions of these neuropeptides are primarily mediated by VPAC1 in this model of PD [141].

Treatment with PACAP showed beneficial effects in mice with experimental autoimmune encephalomyelitis (EAE), by delaying onset of the illness and reducing its severity [142]. The protective effect of PACAP was attributed, at least in part, to its ability to suppress production of proinflammatory mediators such as TNF-*α*, IL-1*β*, and IL-12 in microglia and macrophages and to reduce antigen-presenting efficacy and Th1 differentiation [142].

VIP and PACAP have been largely associated with neuropathic pain [143]. In a rat model of neuropathic pain caused by CCI, blockade of VPAC2 attenuated CCI-induced phosphorylation of p38 and ERK1/2 MAPK in the spinal cord and behavioral reflex sensitization. Conversely, agonist stimulation of VPAC2 in naïve rats increased activation of spinal p38 and ERK1/2 and thermal hyperalgesia [144]. Moreover, intrathecal addition of the glial inhibitor propentofylline reduced thermal hyperalgesia and mechanical allodynia in CCI rats and also reduced MAPK activation in CCI rats and in naïve rats treated with a VPAC2 agonist, suggesting involvement of glial cells in nerve injury-induced sensitization [144]. Astrocytes are known to express VPAC2 (along with VPAC1 and PAC1) [145], and VPAC2 mRNA was shown to be induced upon LPS treatment in mouse peritoneal macrophages [146], THP-1 cells [147], and RAW 264.7 cells [148]. However, expression of VPAC2 has not been detected in microglial cells, either resting or after LPS treatment [60, 61]. In summary, evidence implicates VPAC2-induced MAPK activation in spinal glial cells as a mechanism contributing to sensitization in chronic pain, although specific involvement of microglial cells is yet to be determined.

## 5. Somatostatin

Somatostatin (SST) is a cyclic peptide produced by neuroendocrine, inflammatory, and immune cells, which generally acts as an inhibitor of secretory and proliferative responses in a variety of widely distributed target cells. SST is synthesized from the precursor preprosomatostatin (preproSST), yielding in mammals two bioactive forms termed SST-14 and SST-28. SST receptors are GPCRs, of which five subtypes have been cloned (sst1 to sst5). The five receptors share common signaling pathways, such as inhibition of AC and cAMP formation, induction of tyrosine phosphatase phosphorylation, and modulation of MAPKs through G-protein-dependent mechanisms [149].

In rat primary cultured microglial cells, presence of mRNA for sst2, sst3, and sst4 has been demonstrated [62]. The receptors were functionally active, since SST-14 affected microglial protein phosphorylation and inhibited microglial proliferation induced by granulocyte macrophage colony-stimulating factor (GM-CSF) and IL-3 [62]. Immunoreactivity for sst2, sst3, and sst4 was later detected in BV-2, N9, and mouse primary microglial cells, and SST induced migration in BV-2 and N9 cells [63]. Activation of sst2, sst3, and sst4 in neonatal rat microglia inhibited LPS-induced PGE_2_ production [150], suggesting an anti-inflammatory role for SST in microglial cells. However, a study performed in cortical rat microglia cultures showed no effect of SST on basal or IL-1*β*-induced PGE_2_ synthesis [151], the discrepancies in results being attributed to different experimental settings and to the type of inflammatory stimulus used.

SST stimulates expression of insulin-degrading-enzyme (IDE), an extracellular protease involved in A*β* degradation, in BV-2 and rat microglial cells [152]. Another study showed that SST dose-dependently induces A*β*_1–42_ phagocytosis in BV-2, N9, and rat primary microglial cells, supporting a protective role for SST in the development of AD by modulating microglial function [63]. However, this study showed no effect of SST either alone or in combination with A*β* on IDE or on neprilysin (another enzyme involved in A*β* degradation) expression in BV-2 and N9 cells [63]. The discrepancies may be due to the use in the second study of a much lower concentration of SST (100 nM) versus the 0.5–10 *μ*M concentrations used in the first study or also to differences in incubation times before protein assessment.

In a recent study performed in a rat model of PD generated by injecting LPS into the brain's substantia nigra, SST pretreatment was able to dramatically decrease the number of activated microglial cells [153]. In this study, SST was also able to prevent neuronal cell death and to reduce production of TNF-*α*, IL-1*β*, PGE_2_, and ROS by the substantia nigra [153], all of which are known to be produced by activated microglial cells in models of PD [154]. Therefore, it was suggested that protective effects of SST on neuronal survival in this model of PD are associated with its ability to reduce microglial activation.

Apart from its anti-inflammatory actions STT plays an important role in pain sensitivity. It exerts analgesic effects both centrally and peripherally (reviewed in [155]). However, whether microglial modulation by STT mediates STT-induced analgesia is yet to be determined.

## 6. Cortistatin

Cortistatin (CST) is a cyclic peptide initially isolated from rat brain and named after its predominantly cortical expression and its ability to depress neuronal electrical activity [156]. Its precursor preprocortistatin (preproCST) is a 112-amino acid protein that may suffer proteolytic cleavage at multiple sites, yielding products of different lengths. CST belongs to the same family as SST; the 14-amino acid rat CST isoform and the 17-amino acid human isoform both share 11 amino acids with SST-14, including the motif responsible for SST receptor interaction, and are thereby able to bind all five known SST receptors in vitro with similar affinities to that of SST, acting as receptor agonists and inhibiting cAMP accumulation [157]. Although some of the biological actions of CST seem to be mediated by SST receptors, several effects of CST in the CNS are distinct from those of SST, such as induction of slow-wave sleep and reduction of locomotor activity, suggesting the existence of alternative signaling pathways for CST [157]. In support of this notion, other GPCRs have been shown to bind CST with selective affinity over SST, such as the ghrelin/growth hormone-secretagogue receptor (GHSR) which binds CST with similar affinity compared to ghrelin [158], and the human orphan receptor Mas-related G-protein-coupled receptor member X2 (MrgX2) [159] which also binds proadrenomedullin and related peptides [160]. More recently, truncated functional forms of sst5 have been described which show different signaling profiles in response to CST or SST [161, 162].

Expression of preproCST mRNA in the CNS appears to be restricted to the cortex and hippocampus [156]. In addition to its role as regulator of sleep rhythm and locomotor activity, evidence strongly suggests a role for CST in inflammation and immunomodulation. Expression of preproCST mRNA (but not of preproSST) was detected in human monocyte-derived macrophages and dendritic cells. In addition, CST was demonstrated to bind sst2, and both preproCST and sst2 are upregulated during macrophage differentiation and after LPS stimulation, suggesting CST might be an endogenous ligand for sst2 in the human immune system [163, 164]. In peritoneal macrophages, CST prevented the LPS-induced production of TNF-*α*, IL-12, IL-1*β*, IL-6, NO, MIP-2, and RANTES. Since these effects were only partially prevented by the sst antagonist cyclosomatostatin and were also partially blocked by a GHSR antagonist, evidence suggests CST is acting through sst-dependent and sst-independent pathways in these cells [165, 166]. CST was also shown to dose-dependently inhibit basal and IL-1*β*-induced PGE_2_ release and to reduce IL-1*β*-stimulated COX-2 mRNA expression in primary rat microglial cells [151].

Protective effects of CST have been demonstrated in vivo in experimental models of ulcerative colitis, arthritis, and endotoxemia, where the anti-inflammatory effects of CST were attributed mainly to its ability to deactivate resident and infiltrating macrophages, and other immune cells [165–167]. CST also showed beneficial effects in a model of meningoencephalitis caused by* Klebsiella pneumoniae* infection; it reduced white blood cell infiltration into the cerebrospinal fluid (CSF), attenuated clinical symptoms of illness, inhibited TNF-*α*, IL-1*β*, and IL-6 brain expression and release into the CSF, and decreased neuronal cell death in the cortex and hippocampus [168]. Interestingly, the decrease in* K. pneumoniae*-induced proinflammatory cytokine production after CST treatment was also observed in vitro in neuron-glia cocultures, suggesting direct downregulation of glial activity may partially account for the anti-inflammatory effects of CST [168].

CST also exerted beneficial long lasting effects in models of chronic and relapsing-remitting EAE, where systemic treatment with the neuropeptide reduced incidence and severity of the disease [169]. CST treatment reduced inflammatory spinal cord infiltrates, decreased activation of autoreactive Th1/Th17 cells, and inhibited expression of proinflammatory mediators, while promoting regulatory T cell differentiation. The protective effects of CST were associated with its ability to promote the development of a glial neuroprotective phenotype, by inducing BDNF and activity-dependent neuroprotector protein release from neuron-glial cocultures, reducing astrocyte and microglial IL-6, TNF-*α*, and NO release, and preventing oxidative stress-induced oligodendrocyte death [169].

CST is expressed in interneurons from the spinal cord and in nociceptive neurons from the dorsal root ganglia, where it colocalizes mostly with peptidergic calcitonin gene-related peptide (CGRP)/substance P- (SP-) expressing nociceptors. It is considered to be an endogenous analgesic factor, as nociceptive responses to inflammatory pain are exacerbated in CST knockout mice, and administration of CST induces analgesia and decreases nocifensive behavior in several experimental models of inflammatory pain [170]. However, the analgesic effects of CST appear to be independent of its anti-inflammatory properties and rather exerted by directly inhibiting release of nociceptive peptides such as CGRP and SP from primary nociceptors, mainly through activation of sst2 and GHSR1 [170–172]. Nonetheless, the possibility that CST anti-inflammatory effects on microglial cells may be contributing to its analgesic properties cannot be ruled out.

## 7. Tachykinins

Tachykinins are a family of structurally related peptides derived from proteolytic cleavage of pre-pro-tachykinins, encoded in three* tac* genes and expressed throughout the nervous and immune systems. They regulate many diverse physiological processes including inflammation and nociception and are also involved in many pathological conditions [173]. The major mammalian tachykinins are SP and Neurokinin A (NKA), derived from* tac1,* Neurokinin B (NKB) which is derived from* tac3,* and hemokinin-1 (HK-1), derived from* tac4*. All tachykinins signal through three subtypes of GPCRs termed NK-1R, NK-2R, and NK-3R. NK-1R is the preferential receptor for SP [173]. In humans,* tac4* encodes a precursor protein that has four described splice variants named endokinin (EK) A, EKB, EKC, and EKD. HK-1, EKA, and EKB have SP-like biological actions and can interact with NKRs, whereas EKC and EKD have negligible affinity for NKRs and are considered tachykinin gene-related peptides rather than true tachykinins [173]. Although at first it was believed that* tac4* was expressed only in hematopoietic cells [174], it was later shown that* tac4* displays a broad expression pattern similar to* tac1* and* tac3* [175].

SP has long been recognized as a central and peripheral neuropeptide with stimulatory properties on immune cells. Production of SP and expression of the SP receptor NK-1R were detected in human fetal microglia [64], suggesting that SP can act as an autocrine modulator in these cells. NK-1R protein expression was also detected in M4T.4 and EOC13 microglial cell lines and in primary murine microglia [65]. In keeping with its role as an immune stimulator, SP stimulated IL-6 release in BV-2 cells [176], induced translocation of the NF-*κ*B subunit p65 to the nucleus in M4T.4 cells [65], stimulated thromboxane release [177] and microglial chemotaxis [178], and enhanced LPS-induced IL-1*β* release in rat microglia, although it did not enhance LPS-induced TNF-*α* production or induce cytokine release per se in this study [179]. However, SP was recently shown to increase per se expression of the complement receptor 3, release of TNF-*α* and IL-6, and production of ROS in rat microglial cells, its effects mediated by NK-1R, NK-2R, and NK-3R [66]. SP potentiated class II MHC (MHCII) expression in microglial cells from the brainstem of IFN-*γ*-treated rats, an effect that was prevented by addition of a NK-1R antagonist [180]. However, SP had no significant effect on IFN-*γ*-induced MHCII expression in the hippocampus, suggesting regional differences in microglial response to this neuropeptide [180]. SP also enhanced microglial NF-*κ*B activation, COX-2 expression, PGE_2_ production, and expression of the PGE_2_ receptors EP2 and EP4, induced by* Borrelia burgdorferi*, the causative agent of Lyme disease [181], as well as* B. burgdorferi*- and* Neisseria meningitidis*-induced microglial production of IL-6 and TNF-*α*, in a NK-1R-dependent manner [182].

Evidence suggests that SP may play an important role in the pathogenesis of PD [183]. This neuropeptide is found in particularly high levels in the substantia nigra of the brain [184] and binds NK-1R present in a variety of cells such as endothelial cells, glial cells, and dopaminergic neurons, where it potentiates the release of striatal dopamine [183]. SP was shown to enhance microglial extracellular superoxide and intracellular ROS production through activation of NADPH oxidase, leading to neurotoxicity of dopaminergic neurons [185]. Deletion of endogenous SP in mice was shown to attenuate LPS-induced dopaminergic degeneration, as well as nigral microglial activation and expression of proinflammatory factors [186]. Moreover, addition of SP to microglial cultures potentiated LPS-induced TNF-*α* and nitrite production, MAPK, and NF-*κ*B activation and also enhanced LPS- and 1-methyl-4-phenylpyridinium-induced dopaminergic degeneration in mixed neuron-glia cultures [186].

NK-1R activation has been linked to increased pain sensitivity. In a model of long-term morphine administration, blockade of NK-1R attenuated morphine withdrawal-mediated hyperalgesia and activation of spinal cord microglia [187]. Also, intraperitoneal administration of a NK-1R antagonist alleviated fracture-induced allodynia and reduced spinal cord microglial activation in a rat model of complex regional pain syndrome [188]. In a rat model of spinal sensitization, intrathecal injection of SP induced thermal hyperalgesia and increased p38 phosphorylation in spinal microglial cells; the hyperalgesia was prevented by pretreatment with a p38 inhibitor, supporting a role for microglial p38 activation in nociceptive behavior [189]. In rats suffering from mechanical allodynia, the cytokine TNF-*α*, known to play a key role in neuropathic pain [190], was increased in spinal microglial cells and astrocytes [191]. Concordant with its pronociceptive role, treatment of microglial cells with SP induced production of TNF-*α* mRNA and of transmembrane full-length TNF-*α* (mTNF-*α*), which in turn activates microglia, demonstrated by increased expression of OX-42 and by release of MCP-1, suggesting a possible mechanism through which SP-induced microglial mTNF-*α* expression might create a feed-forward loop and thereby contribute to development of chronic pain [192].

Despite its well-recognized proinflammatory properties, recent reports have suggested an anti-inflammatory and wound healing promoter role for SP. In a rat model of SCI, SP enhanced functional recovery, decreased expression of the M1 markers iNOS, TNF-*α*, and CD86, and induced expression of the M2 markers IL-10, AG1, and CD206 at the injury site. Immunoreactivity of CD206 was colocalized with CD11b^+^ cells in SP-treated animals, suggesting a shift from M1 to M2 microglia/macrophage phenotype [193]. SP also promoted wound healing in a murine model of type 1 diabetes and promoted development of the alternative activation program, determined by a decreased M1/M2 marker ratio in skin macrophages [194].

Expression of* tac4* was detected in a microglial cell line and its levels decreased after incubation with a* B. burgdorferi* lysate [195]. However, another report showed that treatment of rat primary cultured microglia with LPS increased* tac4* mRNA levels through a mechanism involving NF-*κ*B and p38 MAPK [196]. In a rat model of CCI, blockade of NK-2R (and to a lesser extent, NK-1R) attenuated CCI-induced phosphorylation of p38 and ERK1/2 MAPK in the spinal cord and behavioral reflex sensitization. Conversely, treatment of naïve rats with a NK-2R agonist increased activation of spinal p38 and ERK1/2 and induced thermal hyperalgesia. MAPK activation after CCI or after NK-2R activation, as well as CCI-induced behavioral sensitization, was prevented by addition of the glial inhibitor propentofylline [144]. In another rat model of neuropathic pain caused by CCI of the sciatic nerve,* tac4* mRNA was increased in the dorsal horn and blockade of NK-1R prevented this effect and also inhibited associated pain behavior and microglial activation [197]. Altogether, data suggest a role for* tac4* in pathological conditions associated with microglial activation.

## 8. CGRP and Adrenomedullin

CGRP and adrenomedullin (AM) are neuropeptides that belong to the CGRP/calcitonin peptide superfamily, which also includes calcitonin, amylin, and intermedin [198–202]. They are widely distributed in the CNS and PNS and both have potent vasodilator activity [203]. They exert their actions through specific receptors which are GPCRs formed by three components: the 7-transmembrane calcitonin receptor-like receptor (CRLR), the receptor component protein (RCP), and the receptor activity-modifying protein (RAMP) 1–3 [204]. Interaction of CRLR with RAMP1 forms the CGRP1 receptor, which has greater affinity for CGRP than for AM, whereas association with RAMP2 or RAMP3 gives rise to the AM preferring receptors 1 and 2, respectively. CGRP/AM receptor activation is known to stimulate AC and cAMP production, although other signaling pathways have been described such as PLC, intracellular calcium increase, and NO production [203]. Functional evidence indicates microglial cells express CGRP/AM receptors, as treatment with CGRP upregulates immediate-early* c-fos* gene expression and induces cAMP accumulation [67, 68], and both CGRP and AM modulate expression of LPS-induced inflammatory mediators in cultured microglial cells [69].

Cumulative evidence suggests a strong protective anti-inflammatory role for CGRP and AM in several models of diseases, although some proinflammatory effects have also been described for both neuropeptides [205, 206]. Treatment with AM has shown protective effects in a variety of experimental models of disease such as arterial and pulmonary hypertension, heart failure, septic shock, and ischemia-reperfusion injury [207]. AM expression is induced after hypoxia in various cell types of the brain, including neurons, astrocytes, endothelial cells, and microglia [208–210], suggesting a role for the neuropeptide in modulating hypoxic damage in the CNS. In addition, several studies have demonstrated a potent antioxidant role for AM in different cell types, including macrophages [211–214]. In a transient focal ischemia model using AM knockout heterozygous (AM^(+/−)^) mice, the authors showed increased iNOS expression in microglial cells from AM^(+/−)^ mice compared to wild type mice [215]. Furthermore, supernatants of microglial cells exposed to OGD effectively protected neurons against OGD-induced death, and this effect was prevented by an AM receptor antagonist [209], suggesting a neuroprotective role for microglia-derived AM under hypoxic/ischemic stress.

CGRP and AM also inhibit LPS-induced TNF-*α*, IL-6, and NO release in rat cultured microglia and prevent LPS-induced expression of MIP-1*α* and MCP-1 in microglia/astrocyte cocultures. The anti-inflammatory effect appears to be stimulus-specific since neither CGRP nor AM inhibits IL-6 and NO release induced by a cytokine mix composed of TNF-*α*, IFN-*γ*, and IL-1*β* [69]. The ability of neuropeptides to inhibit cytokine and chemokine release in an inflammatory context is of particular relevance in neuroinflammatory diseases such as MS, in which local release of these factors promotes CNS infiltration of leukocytes and autoreactive T cells, which in turn perpetuate and amplify the inflammatory reaction [216]. Concordantly, in a murine model of chronic-progressive EAE, treatment with AM reduced inflammatory infiltration and demyelination in the CNS, partly by impairing activation of autoreactive Th1/Th17 cells and increasing the number of Th2 cells and regulatory T cells and also by preventing oxidative stress-induced oligodendrocyte death and inhibiting expression of astroglial and microglial-derived proinflammatory mediators such as IL-6, IL-12, TNF-*α*, and NO [217]. In another murine model of chronic EAE, infusion of CGRP also decreased clinical signs of disease and prevented microglial activation, evidenced by a reduced proportion of amoeboid Iba1^+^ cells [218]. Altogether, evidence suggests that modulation of microglial activation by CGRP/AM signaling may account, at least in part, for the protective effects of these neuropeptides in neuroinflammatory diseases.

CGRP has been strongly linked to increased pain sensitivity in the spinal cord. Binding of CGRP to CGRP1 receptors in the rat spinal cord produces hyperalgesia through a mechanism involving activation of PKA and PKC signaling pathways [219]. Injection of CGRP stimulates activation of spinal cord microglia in a model of temporomandibular joint disorder, evidenced by increased OX-42 immunostaining [220]. Administration of the CGRP antagonist (CGRP^8–37^) attenuates mechanical hypersensitivity and reduces microgliosis in a rat model of collagen-induced arthritis-induced hypersensitivity [221]. Furthermore, CGRP was demonstrated to contribute to the development of tolerance to morphine-induced analgesia partly through induction of p38 MAPK phosphorylation, upregulation of IL-6, and activation of the NF-*κ*B signaling pathway in spinal cord microglial cells [222–224]. AM is also thought to act as a pronociceptive mediator at the spinal cord level. The pathogenesis of tolerance to chronic morphine treatment is known to involve AM signaling and has been linked to AM-induced proinflammatory activation of spinal microglia and astrocytes [96]. In summary, in experimental models of pain, evidence suggests a strong link between CGRP/AM activity and proinflammatory microglial activation.

## 9. Leptin

Leptin is a cytokine-like 167-amino acid peptide derived from the* ob* gene (or* lep* gene), produced primarily in adipose tissue, and released into the circulation [225]. The concentration of leptin in blood is finely regulated by the nutritional state; it increases following food intake to suppress appetite and decreases with fasting, leptin levels positively correlating with the degree of adiposity in rodents and humans [226, 227]. Given the size of the peptide, central access of leptin to the brain depends mainly on its passage from circulation across the blood-brain barrier (BBB) through a saturable transport system [228]. However, some studies have shown that leptin may also be synthesized in the brain, more specifically in the cerebellum, cortex, and hypothalamus, suggesting specific and local functions for leptin [229].

There are six known isoforms for the leptin receptor (LepR or Ob-R), LepRa-f, which derive from alternative splicing of the* lepr* (or* db*) gene. They all share a common leptin-binding domain but differ in their intracellular domains. LepRe is the only soluble isoform that lacks a transmembrane domain, binds circulating leptin, and inhibits its central transport. LepRb, the long isoform of LepR, is a class I cytokine receptor and the major signaling form of the leptin receptor [225]. Ligand binding to LepRb leads to recruitment and activation of JAK-2, which in turn initiates downstream signaling pathways that may involve MAPKs or different members of the STAT family of transcription factors, such as STAT1, STAT3, and STAT5 [225, 230]. LepRb is widely expressed in the brain, particularly on specialized subsets of neurons in several hypothalamic and brainstem nuclei [231, 232]. In homeostatic conditions, leptin regulates production of POMC, AgRP, and NPY in the ARC [233–235] and inhibits food intake acting at the hypothalamus; it may also modulate neurogenesis, synaptogenesis, neuronal excitability, and neuroprotection in extrahypothalamic sites [236].

Leptin significantly influences the normal functioning of the immune system by stimulating a wide variety of functions such as cytokine production, chemotaxis, cytotoxicity, and survival of immune cells and also plays a role in modulating autoimmune responses in several models of disease (reviewed in [237]). In turn, leptin expression can be regulated by cytokines. Treatment of mice with the proinflammatory mediators TNF-*α* or IL-1 increases serum levels of leptin [238]. Rodent adipose tissue leptin mRNA and circulating levels of leptin are also elevated after systemic LPS injection [238, 239]. Furthermore, LPS-induced fever and loss of appetite were found to be mediated by leptin through induction of IL-1*β* in the brain [240], a finding later confirmed by others [241], all evidence supporting leptin as a mediator of anorexia and cachexia in inflammatory diseases.

The effects of leptin could be due, at least in part, to its actions on microglial cells. Expression of both the short (LepRa) and long (LepRb) isoforms of the leptin receptor was detected in mouse primary cultured glial cells [242] and in rat microglial cells where LepRb is activated by leptin and induces IL-1*β* through a STAT3-mediated mechanism that is independent of caspase-1-mediated cleavage [70]. Leptin treatment of BV-2 cells, which also express LepRa and LepRb, induces IL-6 release by a mechanism involving the insulin-receptor substrate-1/PI3K/Akt/NF-*κ*B signaling pathways [71]. Leptin was also able to induce IL-6 and TNF-*α* release in mouse primary cultured hypothalamic microglia [243]. Preincubation of rat microglial cells with leptin before treatment with LPS potentiated production of IL-1*β*, TNF-*α*, and chemokines such as CINC-1 and MIP-2 [244]. Collectively, data indicate leptin can acutely activate microglial cells, which may be directly involved in the inflammatory effects of leptin in the CNS in pathologic conditions. This effect may be of particular interest in obesity or metabolic syndrome, where production of adipokines is deregulated. In such conditions adipokines can promote inflammation, ROS production, and disruption of BBB permeability and even affect different brain structures like the hippocampus, increasing the risk of developing dementia, such as AD [245]. Also, obesity is known to induce leptin resistance, leading to even higher circulating levels of leptin and further contributing to the pathogenesis of obesity-associated neurodegenerative diseases. In mice with impaired leptin signaling or mice fed a high-fat diet, microglial function is altered or impaired, illustrating a link between adipokines and immunity in the CNS [243]. Moreover, leptin levels are significantly elevated in CSF and in hippocampal tissue of AD patients, and leptin receptor mRNA is decreased, suggesting that leptin signaling is also deregulated in AD brains [246]. Thus, restoration of leptin signaling could result in better functional outcomes in neurodegenerative disease states, especially those associated with obesity and metabolic disorders [247].

Leptin has been shown to contribute to the pathogenesis of neuropathic pain. In a rat model of CCI, administration of a leptin antagonist prevented the development of injury-induced mechanical allodynia and thermal hyperalgesia [248]. In this study, leptin levels were increased in the ipsilateral spinal cord dorsal horn and CSF after CCI, and LepRb expression was also increased in the spinal cord. Interestingly, in vitro exposure of organotypic lumbar spinal cord cultures to leptin induced IL-1*β* production and increased OX-42 immunoreactivity, suggesting spinal microglia as a source of IL-1*β* following leptin treatment [248]. In mice with partial sciatic nerve ligation (PSL), the procedure induced leptin production from adipocytes present in the epineurium of the injured sciatic nerve, and leptin proved to be necessary for PSL-induced tactile allodynia. Furthermore, leptin enhanced production of pronociceptive mediators such as COX-2, iNOS, and matrix metalloprotease-9 from perineural macrophages, linking adipokines to development of neuropathic pain through macrophage activation [249]. However, another report showed protective effects of leptin in a model of SCI, where acute leptin administration after injury enhanced functional motor recovery, prevented development of thermal hyperalgesia and mechanical allodynia, enhanced expression of neuroprotective genes, reduced inflammatory mediators, and decreased spinal cord microglia/macrophage activation [250]. The divergence observed in the effects of leptin between this study and previous ones might be explained by the different experimental models. Thus, further study is needed to clarify the role of leptin in neuropathic pain and the possible involvement of spinal microglial cells as mediators of leptin's action.

## 10. Ghrelin

Ghrelin is a 28-amino acid peptide originally isolated from rat stomach as an endogenous ligand for the GHSR with the ability to stimulate growth hormone (GH) release from the pituitary [251]. It is expressed at high levels in the stomach and is also produced in the ARC of the hypothalamus [251]. Ghrelin mRNA expression in the stomach and circulating plasma levels increase after fasting and decrease after refeeding [252]. It acts as an orexigenic peptide, antagonizing the effects of leptin on food intake through activation of the hypothalamic NPY/Y_1_R pathway [253]. The ghrelin receptor GHSR has two isoforms: GHSR1*α* and GHSR1*β* [254]. Ghrelin acylation in Ser3 is required for hormonal activity and for ghrelin to activate its cognate receptor GHSR1*α* [255], which is expressed in many tissues including pituitary gland and hypothalamus [256]. Nonetheless, unacylated ghrelin is found in circulation at greater concentrations than ghrelin, suggesting a relevant physiological role [255]. Ghrelin can act directly on hypothalamic NPY and AgRP neurons, increasing food intake and body weight gain through a GHSR-dependent, GH-independent pathway [257]. However, certain effects of ghrelin have been observed in tissues where only the GHSR1*β* isoform is expressed, leading to the postulation of the existence of novel nonspecific ghrelin receptors called ghrelin receptor-like receptors and of specific unacylated ghrelin receptors. Finally, GHSR has been shown to form heterodimers with other GPCRs such as sst5, MC3R, and the dopamine receptors D1 and D2. Since receptor heterodimerization can alter G protein coupling as well as ligand potency, these findings add complexity to the pathways involved in ghrelin signaling [254].

Ghrelin has been demonstrated to exert neuroprotective effects in several models of neurodegenerative and inflammatory diseases, in which its protective effects have been associated, at least in part, with its ability to reduce microglial activation. In experimental SCI, microglial p38 MAPK activation followed by pronerve growth factor (proNGF) release is known to mediate oligodendrocyte death [258]. Ghrelin has been shown to promote functional recovery after SCI, partly by inhibiting microglial p38 MAPK activation and proNGF release in the spinal cord and by preventing apoptotic cell death of neurons and oligodendrocytes through a GHSR1*α*-dependent pathway [259]. The inhibitory effects of ghrelin on microglial activation have also been observed in vitro using BV-2 microglial cells. In this assay system, ghrelin prevented LPS-induced BV-2 p38 MAPK and JNK activation and attenuated proNGF and ROS production [260]. However, while expression of GHSR1*α* has been detected in spinal cord neurons and oligodendrocytes, it has not been detected in microglial cells or astrocytes by immunohistochemistry [259] or in BV-2 cells by RT-PCR and western blot [260], suggesting that the mechanism underlying the inhibitory effects of ghrelin on microglial activation may be either indirect or through a GHSR1*α*-independent pathway. In an in vitro assay system of OGD followed by reoxygenation (OGD/RO), modeling SCI, endothelial cell-derived MMP-3 was shown to mediate BV-2 microglial p38 MAPK activation and proNGF release. In this study, supernatant of OGD/RO endothelial cells transfected with MMP-3 siRNA failed to induce BV-2 microglial activation compared to supernatant from cells transfected with control siRNA, and microglial activation was also attenuated in MMP-3 knockout mice. Moreover, addition of ghrelin to OGD/RO endothelial cells inhibited MMP-3 production in a GHSR1*α*-dependent fashion [261], providing a possible mechanism for ghrelin-induced inhibition of microglial activation. In an in vivo model of kainic acid- (KA-) induced hippocampal neurodegeneration, systemic administration of ghrelin prevented KA-induced neuronal cell death, attenuated microglial and astroglial activation, and reduced TNF-*α*, IL-1*β*, and COX-2 expression in the hippocampus. Furthermore, ghrelin inhibited KA-induced MMP-3 expression in hippocampal neurons, and the protective effects of ghrelin were exerted through a GHSR1*α*-dependent pathway [262]. Once again, evidence supports a link between ghrelin-induced modulation of microglial activation and inhibition of MMP-3 expression. Ghrelin also attenuated motoneuron loss in organotypic rat spinal cord cultures exposed to threohydroxyaspartate (THA), a model of excitotoxic motoneuron degeneration, and prevented spinal cord microglia activation and expression of IL-1*β* and TNF-*α*, suggesting a possible therapeutic role for ghrelin in amyotrophic lateral sclerosis [263]. Ghrelin has also shown neuroprotective effects in animal models of PD. In a murine model of MPTP-induced dopaminergic neuron loss, systemic ghrelin administration improved dopaminergic neuron survival, prevented the loss of striatal dopaminergic fibers, and reduced MPTP-induced microglial activation, expression of TNF-*α*, IL-1*β*, MMP-3, and iNOS activation. While the inhibitory effects of ghrelin on microglial activation were prevented by a GHSR1*α* antagonist, expression of GHSR1*α* was not detected either in microglia from the substantia nigra pars compacta or in rat microglia-enriched cultures by immunohistochemistry and RT-PCR, respectively. Therefore, evidence again suggests that ghrelin-mediated inhibition of microglial activation in vivo may be indirect, possibly mediated by the reduction in MMP-3 expression in dopaminergic neurons [264]. In a murine model of EAE, administration of ghrelin reduced clinical signs of disease and inhibited expression of TNF-*α*, IL-1*β*, and IL-6 in spinal cord microglia and in infiltrating T cells from ghrelin-treated mice. The effects of ghrelin on EAE are specific and most likely mediated by GHSR1*α*, as unacylated ghrelin, which lacks the ability to bind GHSR1*α*, had no modulatory effect on EAE. Interestingly, ghrelin also inhibited LPS-induced TNF-*α* release in cultured microglial cells isolated from mouse brain, suggesting ghrelin can directly modulate LPS-induced microglial activation [265], but the specific mechanisms involved in the direct action of ghrelin on microglial cells were not investigated. Ghrelin has shown modulatory effects in memory and learning processes and therefore has been proposed to play a relevant role in AD [266]. CD36 is a scavenger receptor involved in microglial interaction with fibrillar A*β* and a mediator of A*β*-induced microglial activation [72, 73], which also functions as a receptor for GH secretagogues [267]. Bulgarelli and colleagues found that unacylated ghrelin and other synthetic GH secretagogues inhibit A*β*-induced IL-6 and IL-1*β* mRNA expression in N9 microglial cells, whereas acylated ghrelin does not modify A*β*-induced cytokine production in these cells. Moreover, N9 cells express CD36 but do not express GHSR1*α*. Thus, data indicate a role for GH secretagogues other than acylated ghrelin in modulation of A*β*-induced microglial activation through alternative GH receptors, such as CD36 [268]. Altogether, evidence suggests a potential therapeutic role for ghrelin in neurodegenerative disorders involving inflammation and excitotoxic cell death, partly through ghrelin's ability to suppress proinflammatory microglial activation.

Ghrelin has been shown to promote analgesia in models of neuropathic pain. In a rat model of CCI of the sciatic nerve, intrathecal administration of ghrelin delayed mechanical allodynia and thermal hyperalgesia, while reducing p38 MAPK and p65 NF-*κ*B activation, and proinflammatory cytokine expression in the spinal dorsal horn, through a GHSR1*α*-mediated mechanism [269]. Taking into account the ample evidence demonstrating the anti-inflammatory properties of ghrelin, it is possible that its analgesic actions may be due, at least in part, to its microglia-deactivating effect.

## 11. Conclusions

In the past few decades, microglial cells have come to be considered key participants in inflammatory processes within the CNS. They are known to be involved in the development and/or progression of many neuroinflammatory and neurodegenerative diseases, including AD, MS, and PD. Furthermore, research is uncovering an increasingly important role for activated microglia in various neuropsychiatric conditions such as schizophrenia, autism, depression, and anxiety disorders, among others.

The role of microglia in pain sensitivity is a relatively newer area of research, and there is still much to be learnt about how endogenous neuropeptides may influence this phenomenon by acting directly on microglial cells. Nonetheless, it is increasingly evident that these cells are pivotal in development of pathologic pain through the release of inflammatory mediators and neurotransmitters that induce sustained activation of neuronal sensory pathways.

In summary, cumulative evidence suggests a leading role for microglia in pathologies currently of the highest medical relevance. Therefore, the study of central immunomodulatory mediators or microglia-deactivating factors has become a major area of research in the biomedical field, unveiling the existence of precise endogenous mechanisms mediated by neuropeptides that regulate microglial activation, and providing new targets for treatment of neuroinflammatory diseases.

## Figures and Tables

**Table 1:** Expression of neuropeptide receptors in microglial cells.

Receptor	Detection level	Microglial cell system	References
Melanocortin receptors			
MC1R	mRNA	CHME-3 cells	[45]
MC3R	mRNA	CHME-3 cells	[45]
MC4R	mRNA, protein	CHME-3 cells, rat microglia	[45, 47]
MC5R	mRNA	CHME-3 cells	[45]
Opioid receptors			
*μ*-receptors	mRNA, protein	Human fetal microglia, rat microglia	[55, 56]
*κ*-receptors	mRNA, protein	Human fetal microglia, rat microglia	[56, 57]
NPY receptors			
Y_1_R	mRNA, protein	N9 cells, rat retinal microglia	[58, 59]
Y_2_R	mRNA, protein	N9 cells, rat retinal microglia	[58, 59]
Y_5_R	mRNA	N9 cells	[58]
VIP/PACAP receptors			
VPAC1	mRNA	Rat microglia, murine microglia, BV-2, and EOC13 microglial cell lines	[60, 61]
PAC1	mRNA	Rat microglia, murine microglia, BV-2, and EOC13 microglial cell lines	[60, 61]
STT/CST receptors			
sst2	mRNA, protein	Rat microglia, BV-2, N9, and murine microglia	[62, 63]
sst3	mRNA, protein	Rat microglia, BV-2, N9, and murine microglia	[62, 63]
sst4	mRNA, protein	Rat microglia, BV-2, N9, and murine microglia	[62, 63]
Tachykinin receptors			
NK-1R	mRNA, protein	Human fetal microglia, M4T.4, EOC13, and murine microglia	[64, 65]
NK-2R	mRNA	M4T.4 cells	[65]
NK-3R	*Functional evidence*	Rat microglia	[66]
CGRP/AM receptor			
CGRP/AM-R	*Functional evidence*	Rat microglia	[67–69]
Leptin receptors			
LepR	Protein	Rat microglia, BV-2 cells	[70, 71]
LepRa	mRNA	Rat microglia, BV-2 cells	[70, 71]
LepRb	mRNA	Rat microglia, BV-2 cells	[70, 71]
Ghrelin receptors			
CD36 (unacylated ghrelin receptor)	mRNA, Protein	N9 cells, human fetal microglia, and neonatal and adult murine microglia	[72–74]

## References

[1] Franco R., Fernández-Suárez D. (2015). Alternatively activated microglia and macrophages in the central nervous system. *Progress in Neurobiology*.

[2] Gao H.-M., Liu B., Zhang W., Hong J.-S. (2003). Novel anti-inflammatory therapy for Parkinson's disease. *Trends in Pharmacological Sciences*.

[3] McGeer P. L., McGeer E. G. (1995). The inflammatory response system of brain: implications for therapy of Alzheimer and other neurodegenerative diseases. *Brain Research Reviews*.

[4] Minagar A., Shapshak P., Fujimura R., Ownby R., Heyes M., Eisdorfer C. (2002). The role of macrophage/microglia and astrocytes in the pathogenesis of three neurologic disorders: HIV-associated dementia, Alzheimer disease, and multiple sclerosis. *Journal of the Neurological Sciences*.

[5] Frick L. R., Williams K., Pittenger C. (2013). Microglial dysregulation in psychiatric disease. *Clinical and Developmental Immunology*.

[6] Ji R.-R., Berta T., Nedergaard M. (2013). Glia and pain: is chronic pain a gliopathy?. *Pain*.

[7] Ji R.-R., Suter M. R. (2007). p38 MAPK, microglial signaling, and neuropathic pain. *Molecular Pain*.

[8] Ji R.-R., Berta T., Nedergaard M. (2013). Glia and pain: is chronic pain a gliopathy?. *Pain*.

[9] Coull J. A. M., Beggs S., Boudreau D. (2005). BDNF from microglia causes the shift in neuronal anion gradient underlying neuropathic pain. *Nature*.

[10] Ledeboer A., Sloane E. M., Milligan E. D. (2005). Minocycline attenuates mechanical allodynia and proinflammatory cytokine expression in rat models of pain facilitation. *Pain*.

[11] Hains B. C., Waxman S. G. (2006). Activated microglia contribute to the maintenance of chronic pain after spinal cord injury. *The Journal of Neuroscience*.

[12] Chang Y.-W., Waxman S. G. (2010). Minocycline attenuates mechanical allodynia and central sensitization following peripheral second-degree burn injury. *Journal of Pain*.

[13] Pu S., Xu Y., Du D. (2013). Minocycline attenuates mechanical allodynia and expression of spinal NMDA receptor 1 subunit in rat neuropathic pain model. *Journal of Physiology and Biochemistry*.

[14] Hua X.-Y., Svensson C. I., Matsui T., Fitzsimmons B., Yaksh T. L., Webb M. (2005). Intrathecal minocycline attenuates peripheral inflammation-induced hyperalgesia by inhibiting p38 MAPK in spinal microglia. *European Journal of Neuroscience*.

[15] Mika J., Osikowicz M., Makuch W., Przewlocka B. (2007). Minocycline and pentoxifylline attenuate allodynia and hyperalgesia and potentiate the effects of morphine in rat and mouse models of neuropathic pain. *European Journal of Pharmacology*.

[16] Bakos J., Zatkova M., Bacova Z., Ostatnikova D. (2016). The role of hypothalamic neuropeptides in neurogenesis and neuritogenesis. *Neural Plasticity*.

[17] Pocock J. M., Kettenmann H. (2007). Neurotransmitter receptors on microglia. *Trends in Neurosciences*.

[18] Pannell M., Szulzewsky F., Matyash V., Wolf S. A., Kettenmann H. (2014). The subpopulation of microglia sensitive to neurotransmitters/neurohormones is modulated by stimulation with LPS, interferon-*γ*, and IL-4. *Glia*.

[19] Eberle A. N. (1988). *The Melanotropins: Chemistry, Physiology and Mechanisms of Action*.

[20] Wikberg J. E. S., Muceniece R., Mandrika I. (2000). New aspects on the melanocortins and their receptors. *Pharmacological Research*.

[21] Caruso V., Lagerström M. C., Olszewski P. K., Fredriksson R., Schiöth H. B. (2014). Synaptic changes induced by melanocortin signalling. *Nature Reviews Neuroscience*.

[22] Catania A., Gatti S., Colombo G., Lipton J. M. (2004). Targeting melanocortin receptors as a novel strategy to control inflammation. *Pharmacological Reviews*.

[23] Patel H. B., Montero-Melendez T., Greco K. V., Perretti M. (2011). Melanocortin receptors as novel effectors of macrophage responses in inflammation. *Frontiers in Immunology*.

[24] Rajora N., Boccoli G., Burns D., Sharma S., Catania A. P., Lipton J. M. (1997). Alpha-MSH modulates local and circulating tumor necrosis factor-alpha in experimental brain inflammation. *The Journal of Neuroscience*.

[25] Delgado R., Carlin A., Airaghi L. (1998). Melanocortin peptides inhibit production of proinflammatory cytokines and nitric oxide by activated microglia. *Journal of Leukocyte Biology*.

[26] Star R. A., Rajora N., Huang J., Stock R. C., Catania A., Lipton J. M. (1995). Evidence of autocrine modulation of macrophage nitric oxide synthase by *α*-melanocyte-stimulating hormone. *Proceedings of the National Academy of Sciences of the United States of America*.

[27] Rajora N., Ceriani G., Catania A., Star R. A., Murphy M. T., Lipton J. M. (1996). *α*-MSH production, receptors, and influence on neopterin in a human monocyte/macrophage cell line. *Journal of Leukocyte Biology*.

[28] Galimberti D., Baron P., Meda L. (1999). *α*-MSH peptides inhibit production of nitric oxide and tumor necrosis factor-*α* by microglial cells activated with *β*-amyloid and interferon *γ*. *Biochemical and Biophysical Research Communications*.

[29] Giuliani D., Leone S., Mioni C. (2006). Broad therapeutic treatment window of [Nle^4^, D-Phe^7^]*α*-melanocyte-stimulating hormone for long-lasting protection against ischemic stroke, in Mongolian gerbils. *European Journal of Pharmacology*.

[30] Giuliani D., Ottani A., Mioni C. (2007). Neuroprotection in focal cerebral ischemia owing to delayed treatment with melanocortins. *European Journal of Pharmacology*.

[31] Forslin Aronsson Å., Spulber S., Popescu L. M. (2006). *α*-melanocyte-stimulating hormone is neuroprotective in rat global cerebral ischemia. *Neuropeptides*.

[32] Schaible E.-V., Steinsträßer A., Jahn-Eimermacher A. (2013). Single Administration of Tripeptide *α*-MSH(11-13) Attenuates Brain Damage by Reduced Inflammation and Apoptosis after Experimental Traumatic Brain Injury in Mice. *PLoS ONE*.

[33] Lasaga M., Debeljuk L., Durand D., Scimonelli T. N., Caruso C. (2008). Role of *α*-melanocyte stimulating hormone and melanocortin 4 receptor in brain inflammation. *Peptides*.

[34] Caruso C., Carniglia L., Durand D., Scimonelli T. N., Lasaga M., Martins L. M., Loh S. H. Y. (2012). Melanocortins: anti-inflammatory and neuroprotective peptides. *Mental and Behavioural Disorders and Diseases of the Nervous System*.

[35] Roselli-Rehfuss L., Mountjoy K. G., Robbins L. S. (1993). Identification of a receptor for *γ* melanotropin and other proopiomelanocortin peptides in the hypothalamus and limbic system. *Proceedings of the National Academy of Sciences of the United States of America*.

[36] Mountjoy K. G., Mortrud M. T., Low M. J., Simerly R. B., Cone R. D. (1994). Localization of the melanocortin-4 receptor (MC4-R) in neuroendocrine and autonomic control circuits in the brain. *Molecular Endocrinology*.

[37] Bhardwaj R., Becher E., Mahnke K. (1997). Evidence for the differential expression of the functional *α*-melanocyte-stimulating hormone receptor MC-1 on human monocytes. *Journal of Immunology*.

[38] Raap U., Brzoska T., Sohl S. (2003). *α*-melanocyte-stimulating hormone inhibits allergic airway inflammation. *Journal of Immunology*.

[39] Getting S. J., Gibbs L., Clark A. J. L., Flower R. J., Perretti M. (1999). POMC gene-derived peptides activate melanocortin type 3 receptor on murine macrophages, suppress cytokine release, and inhibit neutrophil migration in acute experimental inflammation. *The Journal of Immunology*.

[40] Getting S. J., Christian H. C., Flower R. J., Perretti M. (2002). Activation of melanocortin type 3 receptor as a molecular mechanism for adrenocorticotropic hormone efficacy in gouty arthritis. *Arthritis and Rheumatism*.

[41] Lam C. W., Perretti M., Getting S. J. (2006). Melanocortin receptor signaling in RAW264.7 macrophage cell line. *Peptides*.

[42] Taherzadeh S., Sharma S., Chhajlani V. (1999). *α*-MSH and its receptors in regulation of tumor necrosis factor-*α* production by human monocyte/macrophages. *American Journal of Physiology—Regulatory Integrative and Comparative Physiology*.

[43] Andersen G. N., Hägglund M., Nagaeva O. (2005). Quantitative measurement of the levels of melanocortin receptor subtype 1, 2, 3 and 5 and pro-opio-melanocortin peptide gene expression in subsets of human peripheral blood leucocytes. *Scandinavian Journal of Immunology*.

[44] Lam C. W., Getting S. J., Perretti M. (2005). In vitro and in vivo induction of heme oxygenase 1 in mouse macrophages following melanocortin receptor activation. *The Journal of Immunology*.

[45] Lindberg C., Hjorth E., Post C., Winblad B., Schultzberg M. (2005). Cytokine production by a human microglial cell line: effects of *β*-amyloid and *α*-melanocyte-stimulating hormone. *Neurotoxicity Research*.

[46] Dinulescu D. M., Cone R. D. (2000). Agouti and agouti-related protein: analogies and contrasts. *Journal of Biological Chemistry*.

[47] Carniglia L., Durand D., Caruso C., Lasaga M. (2013). Effect of NDP-*α*-MSH on PPAR-*γ* and –*β* expression and anti-inflammatory cytokine release in rat astrocytes and microglia. *PLoS ONE*.

[48] Taylor A. W. (2005). The immunomodulating neuropeptide alpha-melanocyte-stimulating hormone (*α*-MSH) suppresses LPS-stimulated TLR4 with IRAK-M in macrophages. *Journal of Neuroimmunology*.

[49] Carniglia L., Ramírez D., Durand D. (2016). [Nle4, D-Phe7]-*α*-MSH inhibits toll-like receptor (TLR)2- and TLR4-induced microglial activation and promotes a M2-like phenotype. *PLOS ONE*.

[50] Sarkar A., Sreenivasan Y., Manna S. K. (2003). *α*-Melanocyte-stimulating hormone inhibits lipopolysaccharide-induced biological responses by downregulating CD14 from macrophages. *FEBS Letters*.

[51] Manna S. K., Aggarwal B. B. (1998). *α*-melanocyte-stimulating hormone inhibits the nuclear transcription factor NF-*κ*B activation induced by various inflammatory agents. *Journal of Immunology*.

[52] Mandrika I., Muceniece R., Wikberg J. E. S. (2001). Effects of melanocortin peptides on lipopolysaccharide/interferon-gamma-induced NF-kappaB DNA binding and nitric oxide production in macrophage-like RAW 264.7 cells: evidence for dual mechanisms of action. *Biochemical Pharmacology*.

[53] Taylor A. W., Streilein J. W., Cousins S. W. (1992). Identification of *α*-melanocyte stimulating hormone as a potential immunosuppressive factor in aqueous humor. *Current Eye Research*.

[54] Kawanaka N., Taylor A. W. (2011). Localized retinal neuropeptide regulation of macrophage and microglial cell functionality. *Journal of Neuroimmunology*.

[55] Chao C. C., Hu S., Shark K. B., Sheng W. S., Gekker G., Peterson P. K. (1997). Activation of Mu opioid receptors inhibits microglial cell chemotaxis. *Journal of Pharmacology and Experimental Therapeutics*.

[56] Mika J., Popiolek-Barczyk K., Rojewska E., Makuch W., Starowicz K., Przewlocka B. (2014). Delta-opioid receptor analgesia is independent of microglial activation in a rat model of neuropathic pain. *PLoS ONE*.

[57] Chao C. C., Gekker G., Hu S. (1996). kappa opioid receptors in human microglia downregulate human immunodeficiency virus 1 expression. *Proceedings of the National Academy of Sciences*.

[58] Ferreira R., Xapelli S., Santos T. (2010). Neuropeptide y modulation of interleukin-1*β* (IL-1*β*)-induced nitric oxide production in microglia. *The Journal of Biological Chemistry*.

[59] Santos-Carvalho A., Aveleira C. A., Elvas F., Ambrósio A. F., Cavadas C. (2013). Neuropeptide Y receptors Y_1_ and Y_2_ are present in neurons and glial cells in rat retinal cells in culture. *Investigative Ophthalmology and Visual Science*.

[60] Kim W.-K., Kan Y., Ganea D., Hart R. P., Gozes I., Jonakait G. M. (2000). Vasoactive intestinal peptide and pituitary adenylyl cyclase-activating polypeptide inhibit tumor necrosis factor-*α* production in injured spinal cord and in activated microglia via a cAMP-dependent pathway. *Journal of Neuroscience*.

[61] Delgado M., Jonakait G. M., Ganea D. (2002). Vasoactive intestinal peptide and pituitary adenylate cyclase-activating polypeptide inhibit chemokine production in activated microglia. *Glia*.

[62] Feindt J., Schmidt A., Mentlein R. (1998). Receptors and effects of the inhibitory neuropeptide somatostatin in microglial cells. *Molecular Brain Research*.

[63] Fleisher-Berkovich S., Filipovich-Rimon T., Ben-Shmuel S., Hülsmann C., Kummer M. P., Heneka M. T. (2010). Distinct modulation of microglial amyloid *β* phagocytosis and migration by neuropeptides^i^. *Journal of Neuroinflammation*.

[64] Lai J.-P., Zhan G.-X., Campbell D. E., Douglas S. D., Ho W.-Z. (2000). Detection of substance P and its receptor in human fetal microglia. *Neuroscience*.

[65] Rasley A., Bost K. L., Olson J. K., Miller S. D., Marriott I. (2002). Expression of functional NK-1 receptors in murine microglia. *Glia*.

[66] Zhu J., Qu C., Lu X., Zhang S. (2014). Activation of microglia by histamine and substance P. *Cellular Physiology and Biochemistry*.

[67] Priller J., Haas C. A., Reddington M., Kreutzberg G. W. (1995). Calcitonin gene-related peptide and ATP induce immediate early gene expression in cultured rat microglial cells. *Glia*.

[68] Reddington M., Priller J., Treichel J., Haas C., Kreutzberg G. W. (1995). Astrocytes and microglia as potential targets for calcitonin gene related peptide in the central nervous system. *Canadian Journal of Physiology and Pharmacology*.

[69] Consonni A., Morara S., Codazzi F., Grohovaz F., Zacchetti D. (2011). Inhibition of lipopolysaccharide-induced microglia activation by calcitonin gene related peptide and adrenomedullin. *Molecular and Cellular Neuroscience*.

[70] Pinteaux E., Inoue W., Schmidt L., Molina-Holgado F., Rothwell N. J., Luheshi G. N. (2007). Leptin induces interleukin-1*β* release from rat microglial cells through a caspase 1 independent mechanism. *Journal of Neurochemistry*.

[71] Tang C.-H., Lu D.-Y., Yang R.-S. (2007). Leptin-induced IL-6 production is mediated by leptin receptor, insulin receptor substrate-1, phosphatidylinositol 3-kinase, Akt, NF-*κ*B, and p300 pathway in microglia. *Journal of Immunology*.

[72] Bamberger M. E., Harris M. E., McDonald D. R., Husemann J., Landreth G. E. (2003). A cell surface receptor complex for fibrillar *β*-amyloid mediates microglial activation. *The Journal of Neuroscience*.

[73] Coraci I. S., Husemann J., Berman J. W. (2002). CD36, a class B scavenger receptor, is expressed on microglia in Alzheimer's disease brains and can mediate production of reactive oxygen species in response to *β*-amyloid fibrils. *The American Journal of Pathology*.

[74] El Khoury J. B., Moore K. J., Means T. K. (2003). CD36 mediates the innate host response to *β*-amyloid. *The Journal of Experimental Medicine*.

[75] Sierra A., Abiega O., Shahraz A., Neumann H. (2013). Janus-faced microglia: beneficial and detrimental consequences of microglial phagocytosis. *Frontiers in Cellular Neuroscience*.

[76] Chan A., Magnus T., Gold R. (2001). Phagocytosis of apoptotic inflammatory cells by microglia and modulation by different cytokines: mechanism for removal of apoptotic cells in the inflamed nervous system. *GLIA*.

[77] Durafourt B. A., Moore C. S., Zammit D. A. (2012). Comparison of polarization properties of human adult microglia and blood-derived macrophages. *Glia*.

[78] Montero-Melendez T., Patel H. B., Seed M., Nielsen S., Jonassen T. E. N., Perretti M. (2011). The melanocortin agonist AP214 exerts anti-inflammatory and proresolving properties. *American Journal of Pathology*.

[79] Taylor A. W. (2013). Alpha-melanocyte stimulating hormone (*α*-MSH) is a post-caspase suppressor of apoptosis in RAW 264.7 macrophages. *PLoS ONE*.

[80] Phan T. A., Taylor A. W. (2013). The neuropeptides *α*-MSH and NPY modulate phagocytosis and phagolysosome activation in RAW 264.7 cells. *Journal of Neuroimmunology*.

[81] Starowicz K., Przewłocka B. (2003). The role of melanocortins and their receptors in inflammatory processes, nerve regeneration and nociception. *Life Sciences*.

[82] Tsou K., Khachaturian H., Akil H., Watson S. J. (1986). Immunocytochemical localization of pro-opiomelanocortin-derived peptides in the adult rat spinal cord. *Brain Research*.

[83] Starowicz K., Bilecki W., Sieja A., Przewlocka B., Przewlocki R. (2004). Melanocortin 4 receptor is expressed in the dorsal root ganglions and down-regulated in neuropathic rats. *Neuroscience Letters*.

[84] van der Kraan M., Tatro J. B., Entwistle M. L. (1999). Expression of melanocortin receptors and pro-opiomelanocortin in the rat spinal cord in relation to neurotrophic effects of melanocortins. *Molecular Brain Research*.

[85] Beltramo M., Campanella M., Tarozzo G. (2003). Gene expression profiling of melanocortin system in neuropathic rats supports a role in nociception. *Molecular Brain Research*.

[86] Bertorelli R., Fredduzzi S., Tarozzo G. (2005). Endogenous and exogenous melanocortin antagonists induce anti-allodynic effects in a model of rat neuropathic pain. *Behavioural Brain Research*.

[87] Vrinten D. H., Gispen W. H., Groen G. J., Adan R. A. H. (2000). Antagonism of the melanocortin system reduces cold and mechanical allodynia in mononeuropathic rats. *Journal of Neuroscience*.

[88] Starowicz K., Mousa S. A., Obara I. (2009). Peripheral antinociceptive effects of MC4 receptor antagonists in a rat model of neuropathic pain—A Biochemical and Behavioral Study. *Pharmacological Reports*.

[89] Kalange A. S., Kokare D. M., Singru P. S., Upadhya M. A., Chopde C. T., Subhedar N. K. (2007). Central administration of selective melanocortin 4 receptor antagonist HS014 prevents morphine tolerance and withdrawal hyperalgesia. *Brain Research*.

[90] Inoue K., Tsuda M. (2009). Microglia and neuropathic pain. *GLIA*.

[91] Sacerdote P., Denis Donini S., Paglia P., Granucci F., Panerai A. E., Ricciardicastagnoli P. (1993). Cloned microglial cells but not macrophages synthesize beta-endorphin in response to CRH activation. *Glia*.

[92] Gong N., Xiao Q., Zhu B. (2014). Activation of spinal glucagon-like peptide-1 receptors specifically suppresses pain hypersensitivity. *The Journal of Neuroscience*.

[93] Fan H., Li T.-F., Gong N., Wang Y.-X. (2016). Shanzhiside methylester, the principle effective iridoid glycoside from the analgesic herb *Lamiophlomis rotata*, reduces neuropathic pain by stimulating spinal microglial *β*-endorphin expression. *Neuropharmacology*.

[94] Fan H., Gong N., Li T.-F. (2015). The non-peptide GLP-1 receptor agonist WB4-24 blocks inflammatory nociception by stimulating *β*-endorphin release from spinal microglia. *British Journal of Pharmacology*.

[95] Sundar K. S., Kamaraju L. S., Dingfelder J. (1995). Beta-endorphin enhances the replication of neurotropic human immunodeficiency virus in fetal perivascular microglia. *Journal of Neuroimmunology*.

[96] Zeng X., Lin M. Y., Wang D., Zhang Y., Hong Y. (2014). Involvement of adrenomedullin in spinal glial activation following chronic administration of morphine in rats. *European Journal of Pain (United Kingdom)*.

[97] Raghavendra V., Rutkowski M. D., Deleo J. A. (2002). The role of spinal neuroimmune activation in morphine tolerance/hyperalgesia in neuropathic and sham-operated rats. *Journal of Neuroscience*.

[98] Cui Y., Chen Y., Zhi J.-L., Guo R.-X., Feng J.-Q., Chen P.-X. (2006). Activation of p38 mitogen-activated protein kinase in spinal microglia mediates morphine antinociceptive tolerance. *Brain Research*.

[99] Cui Y., Liao X.-X., Liu W. (2008). A novel role of minocycline: attenuating morphine antinociceptive tolerance by inhibition of p38 MAPK in the activated spinal microglia. *Brain, Behavior, and Immunity*.

[100] Thorsell A., Heilig M. (2002). Diverse functions of neuropeptide Y revealed using genetically modified animals. *Neuropeptides*.

[101] Larhammar D., Salaneck E. (2004). Molecular evolution of NPY receptor subtypes. *Neuropeptides*.

[102] Santos-Carvalho A., Álvaro A. R., Martins J., Ambrósio A. F., Cavadas C. (2014). Emerging novel roles of neuropeptide Y in the retina: from neuromodulation to neuroprotection. *Progress in Neurobiology*.

[103] Ferreira R., Santos T., Cortes L. (2012). Neuropeptide y inhibits interleukin-1 beta-induced microglia motility. *Journal of Neurochemistry*.

[104] Gonçalves J., Ribeiro C. F., Malva J. O., Silva A. P. (2012). Protective role of neuropeptide Y Y_2_ receptors in cell death and microglial response following methamphetamine injury. *European Journal of Neuroscience*.

[105] Vezzani A., Balosso S., Ravizza T. (2008). The role of cytokines in the pathophysiology of epilepsy. *Brain, Behavior, and Immunity*.

[106] Li Q. J., Dong C. Z., Li W. L., Bu W., Wu J., Zhao W. Q. (2014). Neuropeptide Y protects cerebral cortical neurons by regulating microglial immune function. *Neural Regeneration Research*.

[107] Álvaro A. R., Rosmaninho-Salgado J., Santiago A. R. (2007). NPY in rat retina is present in neurons, in endothelial cells and also in microglial and Müller cells. *Neurochemistry International*.

[108] Bedoui S., von Hörsten S., Gebhardt T. (2007). A role for neuropeptide Y (NPY) in phagocytosis: implications for innate and adaptive immunity. *Peptides*.

[109] Ferreira R., Santos T., Viegas M. (2011). Neuropeptide Y inhibits interleukin-1*β*-induced phagocytosis by microglial cells. *Journal of Neuroinflammation*.

[110] Intondi A. B., Dahlgren M. N., Eilers M. A., Taylor B. K. (2008). Intrathecal neuropeptide Y reduces behavioral and molecular markers of inflammatory or neuropathic pain. *Pain*.

[111] Smith P. A., Moran T. D., Abdulla F., Tumber K. K., Taylor B. K. (2007). Spinal mechanisms of NPY analgesia. *Peptides*.

[112] Said S. I., Mutt V. (1970). Polypeptide with broad biological activity: isolation from small intestine. *Science*.

[113] Ganea D., Hooper K. M., Kong W. (2015). The neuropeptide vasoactive intestinal peptide: direct effects on immune cells and involvement in inflammatory and autoimmune diseases. *Acta Physiologica*.

[114] Said S. I. (1986). Vasoactive intestinal peptide. *Journal of Endocrinological Investigation*.

[115] Itoh N., Obata K., Yanaihara N., Okamoto H. (1983). Human preprovasoactive intestinal polypeptide contains a novel PHI-27-like peptide, PHM-27. *Nature*.

[116] Miyata A., Arimura A., Dahl R. R. (1989). Isolation of a novel 38 residue-hypothalamic polypeptide which stimulates adenylate cyclase in pituitary cells. *Biochemical and Biophysical Research Communications*.

[117] Vaudry D., Falluel-Morel A., Bourgault S. (2009). Pituitary adenylate cyclase-activating polypeptide and its receptors: 20 years after the discovery. *Pharmacological Reviews*.

[118] Harmar A. J., Fahrenkrug J., Gozes I. (2012). Pharmacology and functions of receptors for vasoactive intestinal peptide and pituitary adenylate cyclase-activating polypeptide: IUPHAR review 1. *British Journal of Pharmacology*.

[119] Dickson L., Finlayson K. (2009). VPAC and PAC receptors: from ligands to function. *Pharmacology and Therapeutics*.

[120] Waschek J. A. (2013). VIP and PACAP: neuropeptide modulators of CNS inflammation, injury, and repair. *British Journal of Pharmacology*.

[121] Dejda A., Sokołowska P., Nowak J. Z. (2005). Neuroprotective potential of three neuropeptides PACAP, VIP and PHI. *Pharmacological Reports*.

[122] Delgado M., Leceta J., Ganea D. (2003). Vasoactive intestinal peptide and pituitary adenylate cyclase-activating polypeptide inhibit the production of inflammatory mediators by activated microglia. *Journal of Leukocyte Biology*.

[123] Delgado M. (2002). Vasoactive intestinal peptide and pituitary adenylate cyclase-activating polypeptide inhibit CBP-NF-*κ*B interaction in activated microglia. *Biochemical and Biophysical Research Communications*.

[124] Gonzalez-Rey E., Delgado M. (2008). Vasoactive intestinal peptide inhibits cyclooxygenase-2 expression in activated macrophages, microglia, and dendritic cells. *Brain, Behavior, and Immunity*.

[125] Delgado M. (2002). Vasoactive intestinal peptide and pituitary adenylate cyclase-activating polypeptide inhibit the MEKK1/MEK4/JNK signaling pathway in endotoxin-activated microglia. *Biochemical and Biophysical Research Communications*.

[126] Lee H., Suk K. (2004). Selective modulation of microglial signal transduction by PACAP. *NeuroReport*.

[127] Suk K., Park J.-H., Lee W.-H. (2004). Neuropeptide PACAP inhibits hypoxic activation of brain microglia: a protective mechanism against microglial neurotoxicity in ischemia. *Brain Research*.

[128] Delgado M. (2003). Inhibition of interferon (IFN) *γ*-induced Jak-STAT1 activation in microglia by vasoactive intestinal peptide. Inhibitory effect on CD40, IFN-induced protein-10, and inducible nitric-oxide synthase expression. *The Journal of Biological Chemistry*.

[129] Kim W.-K., Ganea D., Jonakait G. M. (2002). Inhibition of microglial CD40 expression by pituitary adenylate cyclase-activating polypeptide is mediated by interleukin-10. *Journal of Neuroimmunology*.

[130] Delgado M., Martinez C., Pozo D. (1999). Vasoactive intestinal peptide (VIP) and pituitary adenylate cyclase-activation polypeptide (PACAP) protect mice from lethal endotoxemia through the inhibition of TNF-*α* and IL-6. *Journal of Immunology*.

[131] Delgado M., Munoz-Elias E. J., Gomariz R. P., Ganea D. (1999). Vasoactive intestinal peptide and pituitary adenylate cyclase-activating polypeptide enhance IL-10 production by murine macrophages: in vitro and in vivo studies. *Journal of Immunology*.

[132] Bhandare A. M., Mohammed S., Pilowsky P. M., Farnham M. M. J. (2015). Antagonism of PACAP or microglia function worsens the cardiovascular consequences of kainic-acid-induced seizures in rats. *Journal of Neuroscience*.

[133] Delgado M. (2003). Vasoactive intestinal peptide prevents activated microglia-induced neurodegeneration under inflammatory conditions: potential therapeutic role in brain trauma. *The FASEB Journal*.

[134] Brifault C., Gras M., Liot D., May V., Vaudry D., Wurtz O. (2015). Delayed pituitary adenylate cyclase-activating polypeptide delivery after brain stroke improves functional recovery by inducing M2 microglia/macrophage polarization. *Stroke*.

[135] Qin X., Sun Z.-Q., Dai X.-J. (2012). Toll-like receptor 4 signaling is involved in PACAP-induced neuroprotection in BV2 microglial cells under OGD/reoxygenation. *Neurological Research*.

[136] Wada Y., Nakamachi T., Endo K. (2013). PACAP attenuates NMDA-induced retinal damage in association with modulation of the microglia/macrophage status into an acquired deactivation subtype. *Journal of Molecular Neuroscience*.

[137] Delgado M., Varela N., Gonzalez-Rey E. (2008). Vasoactive intestinal peptide protects against *β*-amyloid-induced neurodegeneration by inhibiting microglia activation at multiple levels. *Glia*.

[138] Song M., Xiong J.-X., Wang Y.-Y., Tang J., Zhang B., Bai Y. (2012). VIP enhances phagocytosis of fibrillar beta-amyloid by microglia and attenuates amyloid deposition in the brain of APP/PS1 mice. *PLoS ONE*.

[139] Yang S., Yang J., Yang Z. (2006). Pituitary adenylate cyclase-activating polypeptide (PACAP) 38 and PACAP4-6 are neuroprotective through inhibition of NADPH oxidase: Potent regulators of microglia-mediated oxidative stress. *Journal of Pharmacology and Experimental Therapeutics*.

[140] Jenner P. (2003). Oxidative stress in Parkinson's disease. *Annals of Neurology*.

[141] Delgado M., Ganea D. (2003). Neuroprotective effect of vasoactive intestinal peptide (VIP) in a mouse model of Parkinson's disease by blocking microglial activation. *The FASEB Journal*.

[142] Kato H., Ito A., Kawanokuchi J. (2004). Pituitary adenylate cyclase-activating polypeptide (PACAP) ameliorates experimental autoimmune encephalomyelitis by suppressing the functions of antigen presenting cells. *Multiple Sclerosis*.

[143] Dickinson T., Fleetwood-Walker S. M. (1999). VIP and PACAP: very important in pain?. *Trends in Pharmacological Sciences*.

[144] Garry E. M., Delaney A., Blackburn-Munro G. (2005). Activation of p38 and p42/44 MAP kinase in neuropathic pain: involvement of VPAC2 and NK2 receptors and mediation by spinal glia. *Molecular and Cellular Neuroscience*.

[145] Grimaldi M., Cavallaro S. (1999). Functional and molecular diversity of PACAP/VIP receptors in cortical neurons and type I astrocytes. *European Journal of Neuroscience*.

[146] Delgado M., Munoz-Elias E. J., Gomariz R. P., Ganea D. (1999). VIP and PACAP inhibit IL-12 production in LPS-stimulated macrophages. Subsequent effect on IFN*γ* synthesis by T cells. *Journal of Neuroimmunology*.

[147] Delgado M., Ganea D. (2001). Vasoactive intestinal peptide and pituitary adenylate cyclase-activating polypeptide inhibit nuclear factor-*κ*B-dependent gene activation at multiple levels in the human monocytic cell line THP-1. *The Journal of Biological Chemistry*.

[148] Delgado M., Munoz-Elias E. J., Kan Y. (1998). Vasoactive intestinal peptide and pituitary adenylate cyclase-activating polypeptide inhibit tumor necrosis factor *α* transcriptional activation by regulating nuclear factor-kB and cAMP response element-binding protein/c-Jun. *Journal of Biological Chemistry*.

[149] Patel Y. C. (1999). Somatostatin and its receptor family. *Frontiers in Neuroendocrinology*.

[150] Grinshpun J., Tveria L., Fleisher-Berkovich S. (2008). Differential regulation of prostaglandin synthesis in neonatal rat microglia and astrocytes by somatostatin. *European Journal of Pharmacology*.

[151] Dello Russo C., Lisi L., Navarra P., Tringali G. (2009). Diverging effects of cortistatin and somatostatin on the production and release of prostanoids from rat cortical microglia and astrocytes. *Journal of Neuroimmunology*.

[152] Tundo G., Ciaccio C., Sbardella D. (2012). Somatostatin modulates insulin-degrading-enzyme metabolism: implications for the regulation of microglia activity in AD. *PLoS ONE*.

[153] Bai L., Zhang X., Li X. (2015). Somatostatin prevents lipopolysaccharide-induced neurodegeneration in the rat substantia nigra by inhibiting the activation of microglia. *Molecular Medicine Reports*.

[154] More S. V., Kumar H., Kim I. S., Song S.-Y., Choi D.-K. (2013). Cellular and molecular mediators of neuroinflammation in the pathogenesis of Parkinson's disease. *Mediators of Inflammation*.

[155] Pintér E., Helyes Z., Szolcsányi J. (2006). Inhibitory effect of somatostatin on inflammation and nociception. *Pharmacology and Therapeutics*.

[156] De Lecea L., Criado J. R., Prospero-Garcia Ó. (1996). A cortical neuropeptide with neuronal depressant and sleep-modulating properties. *Nature*.

[157] de Lecea L. (2008). Cortistatin—functions in the central nervous system. *Molecular and Cellular Endocrinology*.

[158] Deghenghi R., Papotti M., Ghigo E., Muccioli G. (2001). Cortistatin, but not somatostatin, binds to growth hormone secretagogue (GHS) receptors of human pituitary gland. *Journal of Endocrinological Investigation*.

[159] Robas N., Mead E., Fidock M. (2003). MrgX2 is a high potency cortistatin receptor expressed in dorsal root ganglion. *Journal of Biological Chemistry*.

[160] Kamohara M., Matsuo A., Takasaki J. (2005). Identification of MrgX2 as a human G-protein-coupled receptor for proadrenomedullin N-terminal peptides. *Biochemical and Biophysical Research Communications*.

[161] Córdoba-Chacón J., Gahete M. D., Duran-Prado M. (2010). Identification and characterization of new functional truncated variants of somatostatin receptor subtype 5 in rodents. *Cellular and Molecular Life Sciences*.

[162] Córdoba-Chacón J., Gahete M. D., Durán-Prado M., Luque R. M., Castaño J. P. (2011). Truncated somatostatin receptors as new players in somatostatin-cortistatin pathophysiology. *Annals of the New York Academy of Sciences*.

[163] Dalm V. A. S. H., Van Hagen P. M., Van Koetsveld P. M. (2003). Expression of somatostatin, cortistatin, and somatostatin receptors in human monocytes, macrophages, and dendritic cells. *American Journal of Physiology—Endocrinology and Metabolism*.

[164] Dalm V. A., Van Hagen P. M., Van Koetsveld P. M. (2003). Cortistatin rather than somatostatin as a potential endogenous ligand for somatostatin receptors in the human immune system. *The Journal of Clinical Endocrinology & Metabolism*.

[165] Gonzalez-Rey E., Varela N., Sheibanie A. F., Chorny A., Ganea D., Delgado M. (2006). Cortistatin, an antiinflammatory peptide with therapeutic action in inflammatory bowel disease. *Proceedings of the National Academy of Sciences of the United States of America*.

[166] Gonzalez-Rey E., Chorny A., Robledo G., Delgado M. (2006). Cortistatin, a new antiinflammatory peptide with therapeutic effect on lethal endotoxemia. *The Journal of Experimental Medicine*.

[167] Gonzalez-Rey E., Chorny A., Del Moral R. G., Varela N., Delgado M. (2007). Therapeutic effect of cortistatin on experimental arthritis by downregulating inflammatory and Th1 responses. *Annals of the Rheumatic Diseases*.

[168] Chiu C.-T., Wen L.-L., Pao H.-P., Wang J.-Y. (2011). Cortistatin is induced in brain tissue and exerts neuroprotection in a rat model of bacterial meningoencephalitis. *Journal of Infectious Diseases*.

[169] Souza-Moreira L., Morell M., Delgado-Maroto V. (2013). Paradoxical effect of cortistatin treatment and its deficiency on experimental autoimmune encephalomyelitis. *The Journal of Immunology*.

[170] Morell M., Camprubí-Robles M., Culler M. D., de Lecea L., Delgado M. (2014). Cortistatin attenuates inflammatory pain via spinal and peripheral actions. *Neurobiology of Disease*.

[171] Morell M., Souza-Moreira L., Caro M. (2013). Analgesic effect of the neuropeptide cortistatin in murine models of arthritic inflammatory pain. *Arthritis & Rheumatism*.

[172] Capuano A., Currò D., Navarra P., Tringali G. (2011). Cortistatin modulates calcitonin gene-related peptide release from neuronal tissues of rat. Comparison with somatostatin. *Peptides*.

[173] Steinhoff M. S., von Mentzer B., Geppetti P., Pothoulakis C., Bunnett N. W. (2014). Tachykinins and their receptors: contributions to physiological control and the mechanisms of disease. *Physiological Reviews*.

[174] Zhang Y., Lu L., Furlonger C., Wu G. E., Paige C. J. (2000). Hemokinin is a hematopoietic-specific tachykinin that regulates B lymphopoiesis. *Nature Immunology*.

[175] Kurtz M. M., Wang R., Clements M. K. (2002). Identification, localization and receptor characterization of novel mammalian substance P-like peptides. *Gene*.

[176] Laurenzi M. A., Arcuri C., Rossi R., Marconi P., Bocchini V. (2001). Effects of microenvironment on morphology and function of the microglial cell line BV-2. *Neurochemical Research*.

[177] Giulian D., Corpuz M., Richmond B., Wendt E., Hall E. R. (1996). Activated microglia are the principal glial source of thromboxane in the central nervous system. *Neurochemistry International*.

[178] Maeda K., Nakai M., Maeda S., Kawamata T., Yamaguchi T., Tanaka C. (1997). Possible different mechanism between amyloid-beta (25–35)- and substance P-induced chemotaxis of murine microglia. *Gerontology*.

[179] Martin F. C., Anton P. A., Gornbein J. A., Shanahan F., Merrill J. E. (1993). Production of interleukin-1 by microglia in response to substance P: role for a non-classical NK-1 receptor. *Journal of Neuroimmunology*.

[180] McCluskey L. P., Lampson L. A. (2001). Local immune regulation in the central nervous system by substance P vs. glutamate. *Journal of Neuroimmunology*.

[181] Rasley A., Marriott I., Halberstadt C. R., Bost K. L., Anguita J. (2004). Substance P augments Borrelia burgdorferi-induced prostaglandin E_2_ production by murine microglia. *The Journal of Immunology*.

[182] Chauhan V. S., Sterka D. G., Gray D. L., Bost K. L., Marriott I. (2008). Neurogenic exacerbation of microglial and astrocyte responses to *Neisseria meningitidis* and *Borrelia burgdorferi*. *The Journal of Immunology*.

[183] Thornton E., Vink R. (2015). Substance p and its tachykinin NK1 receptor: a novel neuroprotective target for parkinson’s disease. *Neural Regeneration Research*.

[184] Brownstein M. J., Mroz E. A., Stephen Kizer J., Palkovits M., Leeman S. E. (1976). Regional distribution of substance P in the brain of the rat. *Brain Research*.

[185] Block M. L., Li G., Qin L. (2006). Potent regulation of microglia-derived oxidative stress and dopaminergic neuron survival: substance P vs. dynorphin. *The FASEB Journal*.

[186] Wang Q., Chu C.-H., Qian L. (2014). Substance P exacerbates dopaminergic neurodegeneration through neurokinin-1 receptor-independent activation of microglial NADPH oxidase. *Journal of Neuroscience*.

[187] Tumati S., Largent-Milnes T. M., Keresztes A. I. (2012). Tachykinin NK_1_ receptor antagonist co-administration attenuates opioid withdrawal-mediated spinal microglia and astrocyte activation. *European Journal of Pharmacology*.

[188] Li W.-W., Guo T.-Z., Shi X. (2015). Substance P spinal signaling induces glial activation and nociceptive sensitization after fracture. *Neuroscience*.

[189] Svensson C. I., Marsala M., Westerlund A. (2003). Activation of p38 mitogen-activated protein kinase in spinal microglia is a critical link in inflammation-induced spinal pain processing. *Journal of Neurochemistry*.

[190] Leung L., Cahill C. M. (2010). TNF-*α* and neuropathic pain—a review. *Journal of Neuroinflammation*.

[191] Kiguchi N., Maeda T., Kobayashi Y., Kishioka S. (2008). Up-regulation of tumor necrosis factor-alpha in spinal cord contributes to vincristine-induced mechanical allodynia in mice. *Neuroscience Letters*.

[192] Zhou Z., Peng X., Hagshenas J., Insolera R., Fink D. J., Mata M. (2010). A novel cell-cell signaling by microglial transmembrane TNF*α* with implications for neuropathic pain. *Pain*.

[193] Jiang M. H., Chung E., Chi G. F. (2012). Substance P induces M2-type macrophages after spinal cord injury. *NeuroReport*.

[194] Leal E. C., Carvalho E., Tellechea A. (2015). Substance P promotes wound healing in diabetes by modulating inflammation and macrophage phenotype. *American Journal of Pathology*.

[195] Nelson D. A., Marriott I., Bost K. L. (2004). Expression of hemokinin 1 mRNA by murine dendritic cells. *Journal of Neuroimmunology*.

[196] Sakai A., Takasu K., Sawada M., Suzuki H. (2012). Hemokinin-1 gene expression is upregulated in microglia activated by lipopolysaccharide through NF-*κ*B and p38 MAPK signaling pathways. *PLoS ONE*.

[197] Matsumura T., Sakai A., Nagano M. (2008). Increase in hemokinin-1 mRNA in the spinal cord during the early phase of a neuropathic pain state. *British Journal of Pharmacology*.

[198] Amara S. G., Jonas V., Rosenfeld M. G., Ong E. S., Evans R. M. (1982). Alternative RNA processing in calcitonin gene expression generates mRNAs encoding different polypeptide products. *Nature*.

[199] Roh J., Chang C. L., Bhalla A., Klein C., Hsu S. Y. T. (2004). Intermedin is a calcitonin/calcitonin gene-related peptide family peptide acting through the calcitonin receptor-like receptor/receptor activity-modifying protein receptor complexes. *Journal of Biological Chemistry*.

[200] Kitamura K., Kangawa K., Kawamoto M. (1993). Adrenomedullin: a novel hypotensive peptide isolated from human pheochromocytoma. *Biochemical and Biophysical Research Communications*.

[201] Westermark P., Wernstedt C., Wilander E., Sletten K. (1986). A novel peptide in the calcitonin gene related peptide family as an amyloid fibril protein in the endocrine pancreas. *Biochemical and Biophysical Research Communications*.

[202] Cooper G. J. S., Willis A. C., Clark A., Turner R. C., Sim R. B., Reid K. B. M. (1987). Purification and characterization of a peptide from amyloid-rich pancreases of type 2 diabetic patients. *Proceedings of the National Academy of Sciences of the United States of America*.

[203] Brain S. D., Grant A. D. (2004). Vascular actions of calcitonin gene-related peptide and adrenomedullin. *Physiological Reviews*.

[204] McLatchie L. M., Fraser N. J., Main M. J. (1998). RAMPS regulate the transport and ligand specificity of the calcitonin-receptor-like receptor. *Nature*.

[205] Wong L. Y. F., Cheung B. M. Y., Li Y.-Y., Tang F. (2005). Adrenomedullin is both proinflammatory and antiinflammatory: its effects on gene expression and secretion of cytokines and macrophage migration inhibitory factor in NR8383 macrophage cell line. *Endocrinology*.

[206] Springer J., Geppetti P., Fischer A., Groneberg D. A. (2003). Calcitonin gene-related peptide as inflammatory mediator. *Pulmonary Pharmacology & Therapeutics*.

[207] Bełtowski J., Jamroz A. (2004). Adrenomedullin—what do we know 10 years since its discovery?. *Polish Journal of Pharmacology*.

[208] Ladoux A., Frelin C. (2000). Coordinated up-regulation by hypoxia of adrenomedullin and one of its putative receptors (RDC-1) in cells of the rat blood-brain barrier. *Journal of Biological Chemistry*.

[209] Tixier E., Leconte C., Touzani O., Roussel S., Petit E., Bernaudin M. (2008). Adrenomedullin protects neurons against oxygen glucose deprivation stress in an autocrine and paracrine manner. *Journal of Neurochemistry*.

[210] Bernaudin M., Tang Y., Reilly M., Petit E., Sharp F. R. (2002). Brain genomic response following hypoxia and re-oxygenation in the neonatal rat: identification of genes that might contribute to hypoxia-induced ischemic tolerance. *Journal of Biological Chemistry*.

[211] Oba S., Hino M., Fujita T. (2008). Adrenomedullin protects against oxidative stress-induced podocyte injury as an endogenous antioxidant. *Nephrology Dialysis Transplantation*.

[212] Yoshimoto T., Fukai N., Sato R. (2004). Antioxidant effect of adrenomedullin on angiotensin II-induced reactive oxygen species generation in vascular smooth muscle cells. *Endocrinology*.

[213] Yoshimoto T., Gochou N., Fukai N., Sugiyama T., Shichiri M., Hirata Y. (2005). Adrenomedullin inhibits angiotensin II-induced oxidative stress and gene expression in rat endothelial cells. *Hypertension Research*.

[214] Chini E. N., Chini C. C. S., Bolliger C. (1997). Cytoprotective effects of adrenomedullin in glomerular cell injury: central role of cAMP signaling pathway. *Kidney International*.

[215] Miyamoto N., Tanaka R., Shimosawa T. (2009). Protein kinase A-dependent suppression of reactive oxygen species in transient focal ischemia in adrenomedullin-deficient mice. *Journal of Cerebral Blood Flow & Metabolism*.

[216] Engelhardt B., Ransohoff R. M. (2005). The ins and outs of T-lymphocyte trafficking to the CNS: anatomical sites and molecular mechanisms. *Trends in Immunology*.

[217] Pedreño M., Morell M., Robledo G. (2014). Adrenomedullin protects from experimental autoimmune encephalomyelitis at multiple levels. *Brain, Behavior, and Immunity*.

[218] Sardi C., Zambusi L., Finardi A. (2014). Involvement of calcitonin gene-related peptide and receptor component protein in experimental autoimmune encephalomyelitis. *Journal of Neuroimmunology*.

[219] Sun R.-Q., Tu Y.-J., Lawand N. B., Yan J.-Y., Lin Q., Willis W. D. (2004). Calcitonin gene-related peptide receptor activation produces PKA- and PKC-dependent mechanical hyperalgesia and central sensitization. *Journal of Neurophysiology*.

[220] Cady R. J., Glenn J. R., Smith K. M., Durham P. L. (2011). Calcitonin gene-related peptide promotes cellular changes in trigeminal neurons and glia implicated in peripheral and central sensitization. *Molecular Pain*.

[221] Nieto F. R., Clark A. K., Grist J., Chapman V., Malcangio M. (2015). Calcitonin gene-related peptide-expressing sensory neurons and spinal microglial reactivity contribute to pain states in collagen-induced arthritis. *Arthritis & Rheumatology*.

[222] Wang Z., Ma W., Chabot J.-G., Quirion R. (2010). Morphological evidence for the involvement of microglial p38 activation in CGRP-associated development of morphine antinociceptive tolerance. *Peptides*.

[223] Wang Z., Ma W., Chabot J.-G., Quirion R. (2009). Cell-type specific activation of p38 and ERK mediates calcitonin gene-related peptide involvement in tolerance to morphine-induced analgesia. *FASEB Journal*.

[224] Wang Z., Ma W., Chabot J.-G., Quirion R. (2010). Calcitonin gene-related peptide as a regulator of neuronal CaMKII-CREB, microglial p38-NF*κ*B and astroglial ERK-Stat1/3 cascades mediating the development of tolerance to morphine-induced analgesia. *Pain*.

[225] Münzberg H., Morrison C. D. (2015). Structure, production and signaling of leptin. *Metabolism: Clinical and Experimental*.

[226] Frederich R. C., Hamann A., Anderson S., Lollmann B., Lowell B. B., Flier J. S. (1995). Leptin levels reflect body lipid content in mice: evidence for diet-induced resistance to leptin action. *Nature Medicine*.

[227] Schwartz M. W., Peskind E., Raskind M., Boyko E. J., Porte D. (1996). Cerebrospinal fluid leptin levels: relationship to plasma levels and to adiposity in humans. *Nature Medicine*.

[228] Banks W. A., Kastin A. J., Huang W., Jaspan J. B., Maness L. M. (1996). Leptin enters the brain by a saturable system independent of insulin. *Peptides*.

[229] Morash B., Li A., Murphy P. R., Wilkinson M., Ur E. (1999). Leptin gene expression in the brain and pituitary gland. *Endocrinology*.

[230] Hekerman P., Zeidler J., Bamberg-Lemper S. (2005). Pleiotropy of leptin receptor signalling is defined by distinct roles of the intracellular tyrosines. *The FEBS Journal*.

[231] Elias C. F., Kelly J. F., Lee C. E. (2000). Chemical characterization of leptin-activated neurons in the rat brain. *Journal of Comparative Neurology*.

[232] Scott M. M., Lachey J. L., Sternson S. M. (2009). Leptin targets in the mouse brain. *Journal of Comparative Neurology*.

[233] Schwartz M. W., Seeley R. J., Woods S. C. (1997). Leptin increases hypothalamic pro-opiomelanocortin mRNA expression in the rostral arcuate nucleus. *Diabetes*.

[234] Schwartz M. W., Baskin D. G., Bukowski T. R. (1996). Specificity of leptin action on elevated blood glucose levels and hypothalamic neuropeptide Y gene expression in ob/ob mice. *Diabetes*.

[235] Mizuno T. M., Mobbs C. V. (1999). Hypothalamic agouti-related protein messenger ribonucleic acid is inhibited by leptin and stimulated by fasting. *Endocrinology*.

[236] Bouret S. G. (2010). Neurodevelopmental actions of leptin. *Brain Research*.

[237] La Cava A., Matarese G. (2004). The weight of leptin in immunity. *Nature Reviews Immunology*.

[238] Sarraf P., Frederich R. C., Turner E. M. (1997). Multiple cytokines and acute inflammation raise mouse leptin levels: potential role in inflammatory anorexia. *Journal of Experimental Medicine*.

[239] Grunfeld C., Zhao C., Fuller J. (1996). Endotoxin and cytokines induce expression of leptin, the ob gene product, in hamsters: A role for leptin in the anorexia of infection. *Journal of Clinical Investigation*.

[240] Sachot C., Poole S., Luheshi G. N. (2004). Circulating leptin mediates lipopolysaccharide-induced anorexia and fever in rats. *Journal of Physiology*.

[241] Harden L. M., du Plessis I., Poole S., Laburn H. P. (2006). Interleukin-6 and leptin mediate lipopolysaccharide-induced fever and sickness behavior. *Physiology and Behavior*.

[242] Hosoi T., Okuma Y., Nomura Y. (2000). Expression of leptin receptors and induction of IL-1*β* transcript in glial cells. *Biochemical and Biophysical Research Communications*.

[243] Gao Y., Ottaway N., Schriever S. C. (2014). Hormones and diet, but not body weight, control hypothalamic microglial activity. *GLIA*.

[244] Lafrance V., Inoue W., Kan B., Luheshi G. N. (2010). Leptin modulates cell morphology and cytokine release in microglia. *Brain, Behavior, and Immunity*.

[245] Kiliaan A. J., Arnoldussen I. A. C., Gustafson D. R. (2014). Adipokines: a link between obesity and dementia?. *The Lancet Neurology*.

[246] Bonda D. J., Stone J. G., Torres S. L. (2014). Dysregulation of leptin signaling in Alzheimer disease: evidence for neuronal leptin resistance. *Journal of Neurochemistry*.

[247] Davis C., Mudd J., Hawkins M. (2014). Neuroprotective effects of leptin in the context of obesity and metabolic disorders. *Neurobiology of Disease*.

[248] Lim G., Wang S., Zhang Y., Tian Y., Mao J. (2009). Spinal leptin contributes to the pathogenesis of neuropathic pain in rodents. *Journal of Clinical Investigation*.

[249] Maeda T., Kiguchi N., Kobayashi Y., Ikuta T., Ozaki M., Kishioka S. (2009). Leptin derived from adipocytes in injured peripheral nerves facilitates development of neuropathic pain via macrophage stimulation. *Proceedings of the National Academy of Sciences of the United States of America*.

[250] Fernández-Martos C. M., González P., Rodriguez F. J. (2012). Acute leptin treatment enhances functional recovery after spinal cord injury. *PLoS ONE*.

[251] Kojima M., Hosoda H., Date Y., Nakazato M., Matsuo H., Kangawa K. (1999). Ghrelin is a growth-hormone-releasing acylated peptide from stomach. *Nature*.

[252] Toshinai K., Mondal M. S., Nakazato M. (2001). Upregulation of ghrelin expression in the stomach upon fasting, insulin-induced hypoglycemia, and leptin administration. *Biochemical and Biophysical Research Communications*.

[253] Shintani M., Ogawa Y., Ebihara K. (2001). Rapid publication ghrelin, an endogenous growth hormone secretagogue, is a novel orexigenic peptide that antagonizes leptin action through the activation of hypothalamic neuropeptide Y/Y1 receptor pathway. *Diabetes*.

[254] Callaghan B., Furness J. B. (2014). Novel and conventional receptors for ghrelin, desacyl-ghrelin, and pharmacologically related compounds. *Pharmacological Reviews*.

[255] Hosoda H., Kojima M., Matsuo H., Kangawa K. (2000). Ghrelin and des-acyl ghrelin: two major forms of rat ghrelin peptide in gastrointestinal tissue. *Biochemical and Biophysical Research Communications*.

[256] Guan X.-M., Yu H., Palyha O. C. (1997). Distribution of mRNA encoding the growth hormone secretagogue receptor in brain and peripheral tissues. *Molecular Brain Research*.

[257] Nakazato M., Murakami N., Date Y. (2001). A role for ghrelin in the central regulation of feeding. *Nature*.

[258] Yune T. Y., Lee J. Y., Jung G. Y. (2007). Minocycline alleviates death of oligodendrocytes by inhibiting pro-nerve growth factor production in microglia after spinal cord injury. *The Journal of Neuroscience*.

[259] Lee J. Y., Chung H., Yoo Y. S. (2010). Inhibition of apoptotic cell death by ghrelin improves functional recovery after spinal cord injury. *Endocrinology*.

[260] Lee J. Y., Yune T. Y. (2014). Ghrelin inhibits oligodendrocyte cell death by attenuating microglial activation. *Endocrinology and Metabolism*.

[261] Lee J. Y., Choi H. Y., Yune T. Y. (2015). MMP-3 secreted from endothelial cells of blood vessels after spinal cord injury activates microglia, leading to oligodendrocyte cell death. *Neurobiology of Disease*.

[262] Lee J., Lim E., Kim Y., Li E., Park S. (2010). Ghrelin attenuates kainic acid-induced neuronal cell death in the mouse hippocampus. *Journal of Endocrinology*.

[263] Lee S., Kim Y., Li E., Park S. (2012). Ghrelin protects spinal cord motoneurons against chronic glutamate excitotoxicity by inhibiting microglial activation. *The Korean Journal of Physiology & Pharmacology*.

[264] Moon M., Kim H. G., Hwang L. (2009). Neuroprotective effect of ghrelin in the 1-methyl-4-phenyl-1,2,3,6- tetrahydropyridine mouse model of parkinson's disease by blocking microglial activation. *Neurotoxicity Research*.

[265] Theil M.-M., Miyake S., Mizuno M. (2009). Suppression of experimental autoimmune encephalomyelitis by Ghrelin. *The Journal of Immunology*.

[266] Gahete M. D., Córdoba-Chacón J., Kineman R. D., Luque R. M., Castaño J. P. (2011). Role of ghrelin system in neuroprotection and cognitive functions: implications in Alzheimer's disease. *Peptides*.

[267] Demers A., McNicoll N., Febbraio M. (2004). Identification of the growth hormone-releasing peptide binding site in CD36: a photoaffinity cross-linking study. *Biochemical Journal*.

[268] Bulgarelli I., Tamiazzo L., Bresciani E. (2009). Desacyl-ghrelin and synthetic GH-secretagogues modulate the production of inflammatory cytokines in mouse microglia cells stimulated by *β*-amyloid fibrils. *Journal of Neuroscience Research*.

[269] Zhou C.-H., Li X., Zhu Y.-Z. (2014). Ghrelin alleviates neuropathic pain through GHSR-1a-mediated suppression of the p38 MAPK/NF-*κ*B pathway in a rat chronic constriction injury model. *Regional Anesthesia and Pain Medicine*.

